# Support vector Regression-Optimized Ultrasonic-Assisted alkaline extraction of *Chroogomphus rutilus* Polysaccharides: Enhanced yield and preservation of helical conformations

**DOI:** 10.1016/j.ultsonch.2026.107879

**Published:** 2026-05-03

**Authors:** Xiaorong Zhang, Yufan Zeng, Jiehan Zhang, Weiye Duan, Xinhua Ao, Huizhu Wang, Shuai Chen

**Affiliations:** a College of Chemistry and Pharmaceutical Engineering, Jilin University of Chemical Technology, Jilin 132022, China; b Graduate school, Jilin University of Chemical Technology, Jilin 132022, China

**Keywords:** *Chroogomphus rutilus*polysaccharides, Ultrasound-assisted alkaline extraction, Support vector regression, Response surface methodology, Helical conformation

## Abstract

Polysaccharides from *Chroogomphus rutilus* (CRPs) exhibit promising bioactivities; however, conventional alkaline extraction (CAE) often results in low yields and structural disruption. In this study, a support vector regression (SVR)–optimized ultrasonic-assisted alkaline extraction (UAAE) process was developed to maximize yield while preserving conformational integrity. Compared with the quadratic response surface methodology model, SVR showed superior predictive accuracy and robustness, with the testing *R*^2^ increasing from 0.8751 to 0.9027 and RMSE reduced by 11.7%. SVR also exhibited narrower residual dispersion and improved stability in the high-yield region (>17%), confirming its suitability for nonlinear, multivariable bioprocess optimization. Under the optimized conditions—ultrasonic temperature 56.5 °C, ultrasonic time 35 min, soaking time 138 min, liquid–solid ratio 26 mL/g, NaOH concentration 0.55 mol/L, and ultrasonic power 325 W—the extraction yield reached 20.09 ± 0.10%, representing a 53.7% increase compared with CAE. High-performance gel permeation chromatography revealed two dominant molecular weight (Mw) fractions for UAAE-derived CRP at 7.54 × 10^5^ and 1.32 × 10^4^ Da, whereas CAE-derived products exhibited a trimodal distribution dominated by low-Mw species at 1.43 × 10^3^ Da. Spectroscopic, microscopic, and thermal analyses demonstrated that UAAE more effectively preserved ordered helical conformations, and improved structural stability compared with CAE. CRPs obtained under optimized conditions also showed enhanced antioxidant activity, including ABTS•^+^ and hydroxyl radical scavenging capacities. The integrating acoustic cavitation with alkaline treatment and SVR-based modeling provides an efficient, data-driven strategy for sustainable production of high-quality fungal polysaccharides with preserved bioactive conformations.

## 1 Introduction

*Chroogomphus rutilus* is a rare ectomycorrhizal mushroom that forms symbiotic associations with *Pinus* species in coniferous forests of northeastern and southwestern China [1]. Polysaccharides from *C. rutilus* (CRPs) exhibit immunomodulatory, antioxidant, anti-hyperglycaemic and antitumor activities [2], [3], [4], [5], [6], [7], indicating potential applications in functional foods and pharmaceutical formulations. Effective utilization of these bioactivities requires an extraction strategy that maximizes yield while preserving structural motifs essential for biological function. Fungal polysaccharides display substantial heterogeneity in glycosidic linkages and ordered conformations, including β-glucans and helical structures [8]. These structural features are sensitive to processing conditions and are difficult to preserve during extraction.

Conventional extraction approaches generally rarely achieve both high yield and structural preservation. Hot-water extraction is relatively mild but often inefficient, whereas acid hydrolysis increases yield at the cost of glycosidic integrity. Although alkaline extraction improves polysaccharide solubilization, overly harsh alkaline conditions are often linked to β-elimination–induced depolymerization, structural deterioration, and higher energy consumption. [9]. Ultrasound-assisted extraction (UAE) has emerged as a sustainable and efficient alternative. Acoustic cavitation enhances mass transfer, shortens extraction time, and reduces energy input [10]. When coupled with mild alkaline conditions, ultrasound-assisted alkaline extraction (UAAE) exhibits a synergistic effect on fungal cell wall disassembly, enhanced polysaccharide diffusivity, and overall extraction efficiency. In addition, UAAE has been reported to favor the retention of ordered helical conformations, which are closely associated with polysaccharide bioactivity [11], [12]. Realization of such synergistic effect depends on the concurrent regulation of multiple interdependent parameters, including ultrasonic power, sonication time, NaOH concentration, liquid–solid ratio, and temperature. The resulting response surface is inherently multidimensional and highly nonlinear, rendering conventional low-order polynomial models inadequate for accurate description and prediction [13]. This complexity is reflected in previous studies. For example, *Tenebrio molitor* polysaccharides reached an approximate yield of 9.5% under optimized UAE conditions (75 °C, 150 min, 270 W) [14]. Another study systematically evaluated the effects of ultrasound power, temperature, and particle size on fungal polysaccharide extraction efficiency [15].

Recent advances in machine learning provides effective alternatives to conventional empirical modeling for complex extraction systems [16], [17]. Among these methods, support vector regression (SVR) is particularly suitable for small experimental datasets. By mapping input variables into a high-dimensional feature space using kernel functions, SVR captures nonlinear and interactive effects without explicit variable transformation. Its margin-maximization principle ensures a unique global optimum through convex optimization, while the *ε*-insensitive loss function improves robustness to experimental noise and limits the number of support vectors. SVR therefore yields sparse models with strong generalization performance under limited sampling conditions [18], [19]. Compared with response surface methodology (RSM) or fully connected neural networks, SVR requires fewer hyperparameters, shows reduced susceptibility to local minima, and provides more reliable predictions in sparsely populated design spaces. These properties are particularly advantageous for bioprocess optimization, where experimental measurements are resource-intensive. Machine learning–assisted optimization has been successfully applied to polysaccharide extraction and has outperformed conventional orthogonal experimental designs [20]. For instance, machine learning–based optimization of alginate/guar polysaccharide extraction achieved *R*^2^ values above 0.99 [21]. However, SVR has not yet been applied to optimize the UAAE process for CRPs, and the structural preservation mechanisms under optimized ultrasonic-alkaline conditions remain poorly understood.

This study develops an SVR-based optimization framework for ultrasound-assisted alkaline extraction (UAAE). The sonochemical effects responsible for the improved extraction performance relative to conventional alkaline extraction (CAE) are examined. SVR model is used to describe the nonlinear interactions among five key extraction parameters, and the predicted optimal conditions are experimentally validated. To clarify the role of acoustic cavitation in controlled extraction, a systematic comparative structural analysis is conducted. Structural characteristics, including extraction yield, physicochemical properties, monosaccharide composition, and antioxidant activity, are compared between CRPs obtained under SVR-optimized UAAE conditions and those produced by CAE. By providing an energy-efficient route to high-quality CRPs and offering mechanistic insight into cavitation-regulated extraction behavior, this work presents a practical and generalizable strategy for ultrasonic extraction of bioactive polysaccharides from medicinal fungi.

## 2 Materials and methods

### 2.1 Materials and reagents

Dried fruiting bodies of *Chroogomphus rutilus* were obtained from Lao Dongbei Daisen Wild Specialty Products (Dunhua, China). 1-Phenyl-3-methyl-5-pyrazolone (PMP), 2,2′-azino-bis(3-ethylbenzothiazoline-6-sulfonic acid) (ABTS), and partially methylated alditol acetates (PMAAs) were purchased from Macklin Biochemical Co., Ltd. (Shanghai, China). Monosaccharide standards, including mannose (Man), rhamnose (Rha), glucuronic acid (GlcA), galacturonic acid (GalA), glucose (Glc), galactose (Gal), xylose (Xyl), and fucose (Fuc), were obtained from Sigma-Aldrich (St. Louis, MO, USA). Dialysis tubing (MD44-3500, molecular weight cut-off 3500 Da) was supplied by Hunan Yibo Biotechnology Co., Ltd. (Hunan, China). All other reagents were of analytical grade and used as received. Distilled water was used throughout all experiments.

### 2.2 Comparative extraction methods

Defatted *C. rutilus* (DCR) powder was prepared by refluxing with 95% ethanol (solid–liquid ratio 1:8, w/v) at 65 °C for 4 h to remove lipophilic components. The residue was filtered, washed twice with anhydrous ethanol, and dried to constant weight. For UAAE, DCR was suspended in 0.5 M NaOH (liquid–solid ratio 25:1, v/w) and vortexed for 30 s to ensure homogenization. Ultrasonic treatment was performed using a dual-frequency ultrasonic-microwave-ultraviolet combined catalytic synthesis instrument (Model XH-300UL-2, Beijing Xianghu Technology Development Co., Ltd., China) operating at 25 kHz and equipped with a probe-type titanium alloy horn (20 mm diameter) in continuous mode (100% duty cycle) with adjustable power output (0–1500 W). The probe was immersed 2 cm below the liquid surface to ensure effective cavitation. The mixture was allowed to stand at room temperature for 2 h and centrifuged at 10 000 rpm for 10 min. The supernatant was neutralized to pH 7.0 with 1 mol/L HCl, rested for 10 min, and centrifuged again under identical conditions. The resulting supernatant was dialyzed against distilled water for 48 h using dialysis tubing (MWCO 3500 Da) and subsequently lyophilized to obtain the polysaccharides. For CAE, DCR was mixed with 0.5 mol/L NaOH (liquid–solid ratio 25:1, w/v), vortexed, and extracted under reflux at 100 °C for 2 h. After cooling, the mixture was allowed to stand for 2 h and centrifuged at 10 000 rpm for 10 min. The supernatant was adjusted to pH 7.0, centrifuged again, dialyzed for 48 h (MWCO 3500 Da), and freeze-dried to obtain CRPs.

### 2.3 RSM modeling

To optimize the UAAE process for CRP extraction, a central composite design (CCD) was constructed using Design-Expert v13.0 (Stat-Ease Inc., Minneapolis, MN, USA). Variable ranges were defined based on preliminary single-factor experiments, equipment constraints, and practical processing considerations. Soaking time (*X_1_*, min) was centered on the preliminary optimum (120 min), with bounds ensuring adequate NaOH penetration and enabling evaluation of extended equilibration. NaOH concentration (*X_2_*, mol/L) bracketed the optimum (0.5 mol/L), with the upper limit preventing excessive alkalinity-induced degradation and the lower limit avoiding insufficient cell wall swelling. Ultrasonic time (*X_3_*, min) exceeded the single-factor optimum (30 min) to examine interaction-dependent shifts in optimal duration. Liquid–solid ratio (*X_4_*, mL/g) encompassed the optimum (25 mL/g) while balancing solvent consumption and mass transfer. Ultrasonic power (*X_5_*, W) was limited by equipment capacity and inadequate cavitation below 300 W. Temperature (*X_6_*, °C) was selected to avoid low extraction efficiency below 50 °C and potential polysaccharide instability above 70 °C. These ranges ensured coverage of probable optima within technically feasible operating conditions. Each variable was evaluated at five coded levels (−α, −1, 0, +1, +α), yielding 86 experimental runs (Table 1).

**Table 1:** Independent variables and levels of the CCD.

Independent Variable	Levels				
	−*α*	−1	0	+1	+*α*
Soaking time (min)/*X_1_*	26.1	60	120	180	213.9
NaOH concentration (mol/L)/*X_2_*	0.34	0.4	0.5	0.6	0.66
Liquid-solid ratio (mL/g)/*X_3_*	17.2	20	25	30	32.8
Ultrasonic temperature (°C)/*X_4_*	44.3	50	60	70	75.7
Ultrasonic time (min)/*X_5_*	14.3	20	30	40	45.7
Ultrasonic power (W)/*X_6_*	243.5	300	400	500	556.5

Extraction yield was determined using the phenol–sulfuric acid method [22] and calculated as:

(1) $$\begin{array}{c} Y\left(\%\right)=\frac{C\times V\times D}{M_{0}\times 1000}\times 100 \end{array}$$

where *C* (mg/mL) is the total carbohydrate concentration, *V* (mL) is the filtrate volume, *D* is the dilution factor, and *M_0_* (g) is the mass of DCR powder.

A second-order polynomial model was used to describe the relationship between extraction yield (*Y*) and the six variables (*X_i_*):

(2) $$\begin{array}{c} Y=\beta_{0}+\sum_{i=1}^{6}\beta_{i}X_{i}+\sum_{i=1}^{6}\sum_{j>i}^{6}\beta_{\mathrm{ij}}X_{i}X_{j}+\sum_{i=1}^{6}\beta_{\mathrm{ii}}X_{i}^{2}+\varepsilon \end{array}$$

where *β_0_* is the intercept, *β_i_*, *β_ii_*, and *β_ij_* represent the linear, quadratic, and interaction coefficients, respectively, and ε denotes the error term.

Analysis of variance (ANOVA) was used to assess model significance. Model adequacy was evaluated using *R*^2^, adjusted *R*^2^, predicted *R*^2^, *P* values, and lack-of-fit tests. Three-dimensional response surface and contour plots were generated to visualize variable interactions and identify optimal conditions, which were subsequently validated experimentally.

### 2.4 SVR modeling

#### 2.4.1 Rationale for adopting SVR

Although RSM provides structured experimental design and interpretable polynomial models, it often fails to capture the nonlinearities, synergistic interactions, and threshold effects inherent in UAAE [23]. SVR overcomes these limitations by projecting input variables into a high-dimensional feature space via kernel functions [24], enabling nonlinear modeling without explicit feature engineering. SVR performs well with limited datasets, provides robustness to noise through the ε-insensitive loss function, and guarantees a unique global optimum through convex optimization [24], [25]. Its sparse architecture improves computational efficiency and interpretability. SVR also requires fewer hyperparameters, conforms to Quality-by-Design principles, and has been extensively validated in biochemical process modeling, making it more suitable than RSM for identifying optima in polysaccharide extraction systems [26], [27].

#### 2.4.2 Dataset preparation and preprocessing

The CCD dataset comprising 86 experimental runs (Section 2.3) was used for SVR modeling. Six independent variables—soaking time (*X_1_*, min), NaOH concentration (*X_2_*, mol/L), ultrasonic time (*X_3_*, min), liquid–solid ratio (*X_4_*, mL/g), ultrasonic power (*X_5_*, W), and temperature (*X_6_*, °C)—were used as inputs, and extraction yield (*Y*, %) was the output. The data were randomly stratified into training (80%, n = 69) and testing (20%, n = 17) subsets, ensuring balanced yield distribution across quintiles. No outliers were detected (Mahalanobis *D*^2^ < χ^2^_0.999_(6) = 22.46). All variables were normalized to a [0,1] scale using Min–Max scaling:

(3) $$\begin{array}{c} x_{\mathrm{norm}}=\frac{x-x_{\min}}{x_{\max}-x_{\min}} \end{array}$$

**Fig. 1:**
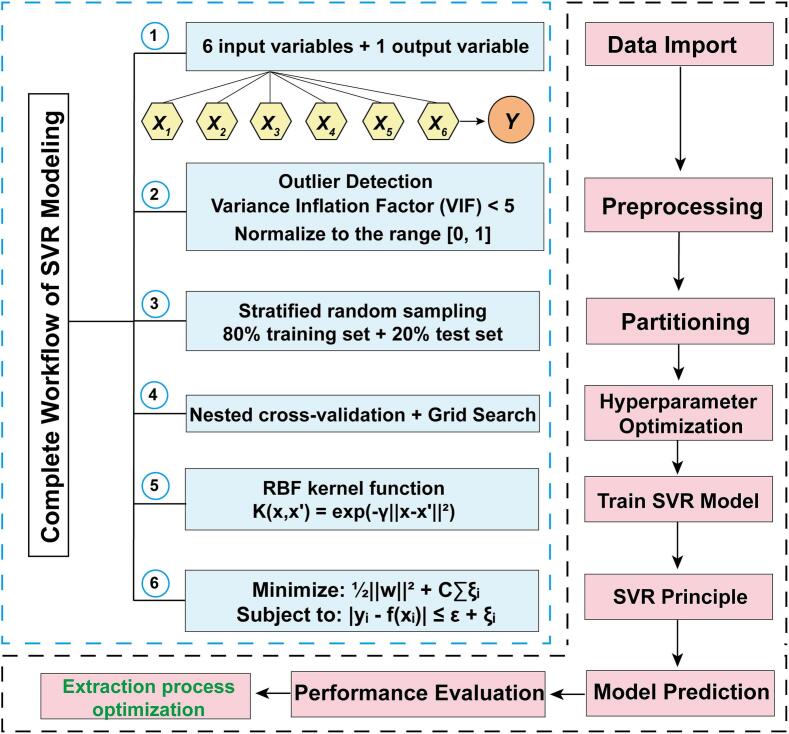
Complete workflow of SVR modeling for extraction process optimization.

Variance inflation factors (VIFs = 1.23–3.87 < 5) indicated negligible multicollinearity. The integrated workflow—outlier screening, normalization, and stratified partitioning—ensured balanced feature contributions and fair model comparison (Fig. 1).

#### 2.4.3 SVR model architecture and mathematical formulation

An SVR model with a radial basis function (RBF) kernel was constructed to map the relationship between process variables and extraction yield. For training data {(*X_i_*, *Y_i_*)}^n^_(i=1)_, the SVR optimization objective is:

(4) $$\begin{array}{c} \min_{w,b,\xi }\frac{1}{2}{\left\|w\right\|}^{2}+C\sum_{i=1}^{n}\xi_{i}+\xi_{i}^{*} \end{array}$$

subject to

(5) $$\begin{array}{c} \left\{\begin{array}{c} Y_{i}-\left(w^{T}\phi \left(X_{i}\right)+b\right)\leq \varepsilon +\xi_{i}\\ \left(W^{T}\phi \left(X_{i}\right)+b\right)-Y_{i}\leq \varepsilon +{\xi_{i}}^{*}\\ \xi_{i},{\xi_{i}}^{*}\geq 0 \end{array}\right) \end{array}$$

The resulting dual form leads to the following regression function:

(6) $$\begin{array}{c} f\left(X\right)=\sum_{i=1}^{n_{\mathrm{sv}}}\left(\alpha_{i}-\alpha_{i}^{*}\right)K\left(X,X_{i}\right)+b \end{array}$$

where α_i_, α_i_^∗^ represent Lagrange multipliers. The RBF kernel was defined as:

(7) $$\begin{array}{c} K\left(X,X^{\prime }\right)=\exp\left(-\gamma {\|X-X^{\prime }\|}^{2}\right)=\exp\left(-\frac{{\|X-X^{\prime }\|}^{2}}{2\delta^{2}}\right) \end{array}$$

with *γ* = 1/(2*σ*^2^) controlling kernel width.

#### 2.4.4 Hyperparameter optimization strategy

Three parameters—penalty coefficient (*C*), kernel width (*γ*), and *ε*—were optimized using grid search combined with nested five-fold cross-validation. The inner loop performed parameter tuning, whereas the outer loop evaluated generalization. Grid ranges were *C* = {0.1, 1, 10, 100, 1000}, *γ* = {0.001, 0.01, 0.1, 1, 10}, and *ε* = {0.001, 0.01, 0.1, 1.0}, yielding 100 combinations per fold. Optimal parameters minimized the cross-validation RMSE. Final values were averaged across folds to reduce random variation.

#### 2.4.5 Model performance evaluation metrics

SVR predictive performance was evaluated using standard indices:

(8) $$\begin{array}{c} R^{2}=1-\frac{\sum_{i=1}^{n}{\left(y_{i}-{\hat{y}}_{i}\right)}^{2}}{\sum_{i=1}^{n}{\left(y_{i}-\overset{¯}{y}\right)}^{2}} \end{array}$$

(9) $$\begin{array}{c} \mathrm{RMSE}=\sqrt{\frac{1}{n}\sum_{i=1}^{n}{\left(y_{i}-{\hat{y}}_{i}\right)}^{2}} \end{array}$$

(10) $$\begin{array}{c} \mathrm{MAE}=\frac{1}{n}\sum_{i=1}^{n}\left|y_{i}-{\hat{y}}_{i}\right| \end{array}$$

(11) $$\begin{array}{c} \mathrm{MAPE}=\frac{1}{n}\sum_{n=1}^{n}\left|\frac{y_{i}-{\hat{y}}_{i}}{y_{i}}\right| \end{array}$$

Generalization stability was assessed using Δ*R*^2^ = *R*^2^_train_ – *R*^2^_test_ (acceptable if < 0.10). A paired *t*-test (*α* = 0.05, *n* = 17) was employed to compare absolute prediction errors between SVR and RSM. Residual diagnostics confirmed model adequacy: normality (Lilliefors, Jarque–Bera, Anderson–Darling; *P* > 0.05), homoscedasticity (Breusch–Pagan; *P* > 0.05), and independence (Durbin–Watson ≈ 2).

#### 2.4.6 Sensitivity analysis and feature importance

Three complementary methods were used to quantify variable importance.

Permutation importance (PI) was calculated as follows:

(12) $$\begin{array}{c} PI_{j}=\frac{{\mathrm{RMSE}}_{j}-{\mathrm{RMSE}}_{0}}{{\mathrm{RMSE}}_{0}} \end{array}$$

Partial dependence (PD) was calculated as follows:

(13) $$\begin{array}{c} {\mathrm{PD}}_{j}\left(x_{j}\right)=\frac{1}{n}\sum_{i=1}^{n}\hat{f}\left(x_{j},X_{i,-j}\right) \end{array}$$

Dynamic range (PDR) normalization enabled comparability across variables.

Support vector contribution (SV) was calculated as follows:

(14) $$\begin{array}{c} {\mathrm{SV}}_{j}=\frac{\sum_{i\in SV}\left(\left|a_{i}\right|-\left|x_{\mathrm{ij}}\right|\right)}{\sum_{j}\sum_{i\in SV}\left(\left|a_{i}\right|-\left|x_{\mathrm{ij}}\right|\right)} \end{array}$$

The three scores (PI, PD, SV) were normalized to [1] and integrated via weighted averaging (0.50, 0.25, 0.25) to yield a unified importance ranking.

#### 2.4.7 Process optimization and experimental validation

The optimized SVR model was used to identify the maximum polysaccharide yield within the CCD-defined ranges: soaking time 60–180 min, NaOH 0.4–0.6 mol/L, ultrasonic time 40–60 min, liquid–solid ratio 20–30 mL/g, ultrasonic power 300–500 W, and temperature 50–70 °C. Owing to the model’s sparsity and kernel efficiency, the optimal solution was obtained within seconds. Theoretical optima were adjusted to practical experimental tolerances (±5 min, ±0.05  mol/L) and validated in triplicate (*n* = 3). Agreement between predicted and observed yields was evaluated using absolute and relative error within 95% prediction intervals. Comparative experiments under RSM-optimized and CCD-center conditions were conducted, and statistical significance was assessed by one-way ANOVA followed by Tukey’s HSD test (α = 0.05).

### 2.5 Economic comparison of extraction methods

#### 2.5.1 Production rate

Production rate (PR), defined as the polysaccharide yield per unit time, was calculated as:

(15) $$\begin{array}{c} PR\left(\%/h\right)=\frac{Y}{t} \end{array}$$

where *Y* is the extraction yield (%) and *t* is the extraction time (h).

#### 2.5.2 Specific energy consumption

Specific energy consumption (SEC), defined as the energy required to produce 1 kg of CRP, was calculated as:

(16) $$\begin{array}{c} \mathrm{SEC}\left(kWh/kg\right)=\frac{P\times t}{m} \end{array}$$

where *P* is the operating power of the extraction equipment (kW), including ultrasonic power for UAAE-based processes and heating power for conventional water-bath extraction, *t* is the extraction time (h), and *m* is the mass of extracted polysaccharide (kg).

#### 2.5.3 CO_2_ emissions

Environmental impact was assessed based on CO_2_ emissions, and total emissions were calculated as:

(17) $$\begin{array}{c} {\mathrm{CO}}_{2}emissions\left(kg{\mathrm{CO}}_{2}/kgpolysaccharide\right)=SEC\times EF \end{array}$$

where SEC is the specific energy consumption (kWh/kg polysaccharide) and EF is the electricity carbon emission factor (kg CO_2_/kWh). The emission factor was set at 0.4671 kg CO_2_ /kWh, according to the province-specific average electricity carbon dioxide emission factor for 2023 reported by the Ministry of Ecology and Environment of the People's Republic of China [28].

This assessment integrates energy consumption and emission intensity, enabling a comprehensive evaluation of extraction sustainability.

### 2.6 Characterization of raw materials before and after extraction

#### 2.6.1 Fourier transform infrared (FT-IR) spectroscopy

Dried *C. rutilus* powder was finely ground and homogenized with spectroscopic-grade KBr at a ratio of 1:100 (w/w), further ground in an agate mortar for 5 min, and compressed into pellets under 740 MPa (10 t, 13 mm die) for 30 s. FT-IR spectra were collected using a Nicolet 6700 spectrometer (Thermo Fisher Scientific, Waltham, MA, USA) over the range of 4000–400 cm⁻^1^.

#### 2.6.2 Scanning electron microscopy (SEM)

Approximately 5 mg of dried sample was affixed onto conductive carbon tape and sputter-coated with gold for 40 s. Microstructural observation was performed using an ultra-high-resolution scanning electron microscope (SU8600, Hitachi, Tokyo, Japan) at an accelerating voltage of 5 kV.

#### 2.6.3 X-ray diffraction (XRD)

Crystallinity was analyzed using a D8 Advance diffractometer (Bruker AXS, Karlsruhe, Germany) equipped with Cu Kα radiation (λ = 1.5406 Å), operated at 40 kV and 15 mA. Diffraction patterns were recorded over a 2θ range of 5–90°.

#### 2.6.4 Thermogravimetric analysis (TGA)

Thermal stability was evaluated using a simultaneous thermal analyzer (SDT650, TA Instruments, New Castle, DE, USA). Approximately 1–2 mg of sample was placed in an aluminum pan and heated from 50 to 600 °C at a rate of 10 °C/min under nitrogen flow (50 mL/min). Thermogravimetric (TG) and derivative thermogravimetric (DTG) curves were recorded, and onset, peak, and completion temperatures of each mass-loss stage were determined from DTG profiles.

### 2.7 Physicochemical properties of CRP extracted by different methods

#### 2.7.1 Chemical composition analysis

Total sugar, uronic acid, and protein contents of CRP were determined. Total polysaccharides were quantified using the phenol–sulfuric acid method. Briefly, 1.0 mL of sample solution was mixed with 1.0 mL of 5% (w/v) phenol and 5.0 mL of concentrated sulfuric acid. After vortexing, the mixture was incubated at 40 °C for 30 min, cooled to room temperature, and absorbance was measured at 490 nm using a UV–Vis spectrophotometer (TU-1950, Beijing Puxi General Instruments, China). A glucose standard curve (*Y* = 50.64*X* − 0.0046, *R*^2^ = 0.9995) was used for quantification. Uronic acid content was determined by the sulfuric acid–carbazole method using galacturonic acid as the standard. After heating and color development, samples were reacted with carbazole and measured at 530 nm, with a calibration curve of *Y* = 0.0303*X* − 0.0543, (*R*^2^ = 0.9997). Protein content was determined using a BCA assay kit (Beyotime Biotechnology, Shanghai, China). Samples (20 μL) were mixed with BCA reagent (200 μL) and incubated at 37 °C for 30 min in a 96-well plate. Absorbance was measured at 562 nm, with BSA as the standard (*Y* = 0.0415*X* + 0.0245, *R*^2^ = 0.9996).

#### 2.7.2 Monosaccharide composition determination

CRP (10 mg) was hydrolyzed with 2 mol/L TFA (7 mL) at 110 °C for 5 h. After centrifugation, residual TFA was removed by co-evaporation with methanol. An aliquot of hydrolysate (100 μL) was derivatized with PMP in the presence of NaOH, incubated at 70 °C for 60 min, neutralized with HCl, and extracted with chloroform. The aqueous phase was diluted to 2 mL, filtered (0.45 μm), and analyzed by HPLC. Chromatographic separation was performed using an Agilent 1260 HPLC system equipped with a ZORBAX Eclipse XDB-C_18_ column (4.6 × 250  mm, 5  μm). The mobile phase comprised 0.05  mol/L KH_2_PO_4_ containing 18% acetonitrile at a flow rate of 1.0 mL/min. Detection was conducted at 245 nm. Standards included Man, Rha, GlcA, GalA, Glc, Gal, Xyl, and Fuc, all with R^2^ > 0.999.

#### 2.7.3 Molecular weight distribution

Molecular weight distribution was determined by high-performance gel permeation chromatography (HPGPC). CRP samples and standards (5 mg/mL) were sonicated, centrifuged (12 000 rpm, 10 min), and filtered through a 0.22-μm membrane. Analysis was performed using a Thermo UltiMate 3000 system equipped with a Shimadzu RI-20A detector and BRT 105–103–101 columns. The mobile phase was 0.2 mol/L NaCl at a flow rate of 0.8 mL/min, 40 °C, and injection volume 25 μL.

#### 2.7.4 UV–Vis spectroscopy

Residual proteins and nucleic acids were evaluated by UV–Vis spectroscopy (Beijing Purkinje, China). CRP samples (5 mg) was dissolved to 0.5 mg/mL and scanned from 200 to 600 nm against a water blank.

#### 2.7.5 Atomic force microscopy (AFM)

Surface microtopography was examined by AFM. CRP samples (10 μg/mL) prepared in ultrapure water were deposited (50 μL) onto freshly cleaved mica, dried under nitrogen, and imaged using a Dimension FastScan microscope (Bruker, USA) in tapping mode. Scan sizes ranged from 1 to 5 μm at a resolution of 512 × 512 pixels and scan rates of 1–2 Hz.

#### 2.7.6 Congo red test

The Congo red assay was used to assess helical conformations. Briefly, 2.0 mL of CRP solution (2 mg/mL) was mixed with 2.0 mL of Congo red solution (80 μmol/L). Subsequently, 1.0 mL of NaOH solution (0, 0.5, 1.0, 1.5, 2.0, 2.5, or 3.0 mol/L) was added to obtain final NaOH concentrations of 0–0.6 mol/L. After incubation at 25 °C for 30 min, absorption spectra were recorded using a TU-1950 UV–Vis spectrophotometer (Beijing Puxi General Instruments Co., Ltd., China) over 200–800 nm. The maximum absorption wavelength (*λ*_max_) was determined at each NaOH concentration. A plot of *λ*_max_ versus NaOH concentration was used to evaluate the stability of the CRP–Congo red complex.

#### 2.7.7 Particle size and zeta potential

Particle size and zeta potential were measured using a Zetasizer Nano ZS90 (Malvern Panalytical, UK) equipped with a 633 nm He–Ne laser (173° backscatter). CRP solutions (0.1 mg/mL) were filtered (0.45 μm) and analyzed at 25 ± 0.1 °C. Dynamic light scattering provided the Z-average diameter and polydispersity index (PDI), while zeta potential was measured using a folded capillary cell (DTS1070) and calculated using the Smoluchowski equation. Results are expressed as mean ± SD from triplicate measurements.

#### 2.7.8 Rheological properties

Rheological properties were measured using a DHR-2 rheometer (TA Instruments, USA) fitted with a 20 mm parallel plate geometry (gap 1000 μm). CRP solutions (50 mg/mL) were equilibrated at 25 ± 0.1 °C. Shear viscosity was recorded over shear rates of 0.1–1000 s⁻^1^. Dynamic viscoelastic parameters (G′ and G″) were obtained from frequency sweeps (0.1–100 rad/s) within the linear viscoelastic region, determined by strain sweeps (0.01–100% at 1 rad/s). Yield stress was obtained from stress ramp measurements (0.1–1000 Pa).

#### 2.7.9 Other property measurements

FT-IR, SEM, XRD, and TGA analyses of CRPs were performed following the procedures described in Section 2.6.

### 2.8 In vitro biological activities of CRP

#### 2.8.1 ABTS radical cation scavenging assay

ABTS•⁺ was generated by mixing 7 mmol/L ABTS with 2.45 mmol/L potassium persulfate and incubating the mixture in the dark at room temperature for 14 h. The resulting solution was diluted with PBS (pH 7.4) to an absorbance of 0.70 ± 0.02 at 734 nm. CRPs sample (2 mL) was mixed with ABTS•⁺ solution (2 mL), incubated at room temperature for 5 min, and absorbance was measured at 734 nm. Ascorbic acid (Vc) was used as the positive control. Scavenging activity was calculated as:

(18) $$\begin{array}{c} scavengingrate\left(\%\right)=\left[1-\frac{\left(A_{s}-A_{0}\right)}{\left(A_{b}-A_{0}\right)}\right]\times 100 \end{array}$$

where *A_s_* is the absorbance of the sample, *A_b_* is the control (sample replaced with water). and *A_0_* is the blank (without ABTS•⁺)

#### 2.8.2 Hydroxyl radical scavenging assay

Hydroxyl radical (•OH) scavenging activity was evaluated using the o-phenanthroline–Fe^2+^–H_2_O_2_ system. The reaction mixture consisted of o-phenanthroline (1 mL, 2.5 mmol/L), PBS (2 mL), water (1 mL), ferrous sulfate (1 mL, 2.5 mmol/L), and H_2_O_2_ (1 mL, 0.077%). After incubation at 37 °C for 60 min, absorbance was recorded at 536  nm (*A_1_*). Controls included *A_0_* (H_2_O_2_ omitted) and *A_s_* (water replaced with CRPs solution). Vc was used as the positive control. Scavenging activity was calculated according to Eq. (18).

### 2.9 Data analysis

All analyses were performed using OriginPro 2024 (OriginLab, USA) and GraphPad Prism 9 (GraphPad Software, USA). Group comparisons were conducted using one-way ANOVA followed by Tukey’s post hoc test. Data normality and homogeneity of variance were verified using the Shapiro–Wilk and Levene’s tests (*P* > 0.05). Results are presented as mean ± SD. Unless otherwise stated, all statistical tests were two-tailed, with significance thresholds of at *P* < 0.05, *P* < 0.01, and *P* < 0.001.

## 3 Results and discussion

### 3.1 Comparative optimization by RSM

#### 3.1.1 Influence of single factor extraction parameters

**Fig. 2:**
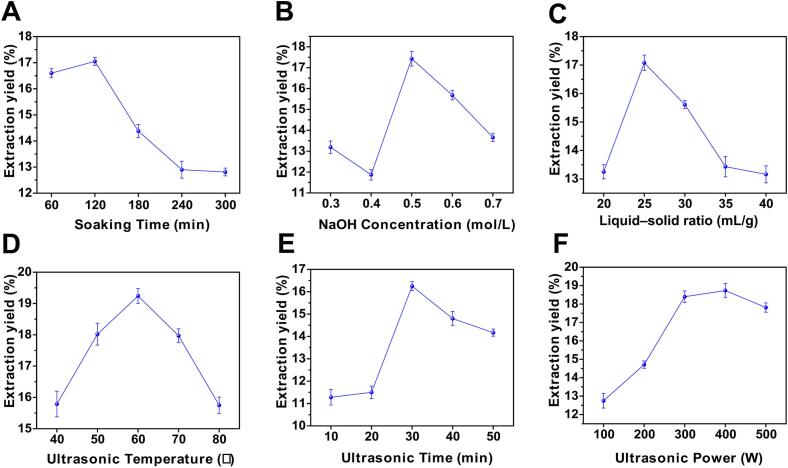
Extraction yields of CRP under different single-factor conditions: (A) soaking time, (B) NaOH concentration, (C) ultrasonic time, (D) liquid–solid ratio, (E) ultrasonic power, and (F) ultrasonic temperature.

As shown in Fig. 2, all investigated parameters significantly influenced CRP extraction yield. Soaking time increased the yield up to 120 min (17.05%) and declined thereafter, likely due to polysaccharide degradation or re-dissolution [29]. Similarly, ultrasonic duration up to 30 min enhanced yield (16.25%), followed by a decrease at longer times. The liquid–solid ratio exhibited a bell-shaped trend, with a maximum yield (17.08%) at 25 mL/g; both lower and higher ratios reduced efficiency because of solvent limitation or dilution effects [12]. NaOH concentration peaked at 0.5 mol/L (17.43%), beyond which excessive alkalinity likely induced cell wall collapse or polysaccharide degradation [30]. Ultrasound power improved yield up to 400 W (18.73%), whereas excessive power (500 W) reduced yield, possibly due to chain scission caused by cavitation collapse [31]. Extraction temperature showed a similar pattern, with a maximum at 60 °C (19.24%) followed by a decline, reflecting the thermal sensitivity of the polysaccharide matrix [29]. The optimal single-factor conditions were therefore identified as soaking time 120 min, ultrasonic time 30 min, liquid–solid ratio 25 mL/g, NaOH concentration 0.5 mol/L, ultrasonic power 400 W, and ultrasonic temperature 60 °C.

#### 3.1.2 Model fitting and statistical analysis

The experimental design and results of the RSM optimization are presented in Table S1. Data were analyzed using Design-Expert 13 software, yielding a quadratic polynomial model for extraction yield (*Y*) as a function of the coded variables (*X_1_*–*X_6_*):

(19) $$\begin{array}{c} Y=19.33+0.3460X_{1}+0.1802X_{2}+0.5011X_{3}+0.4286X_{4}+0.2081X_{5}-0.2556X_{6}\\ +0.0886X_{1}X_{2}-0.0067X_{1}X_{3}+0.0236X_{1}X_{4}+0.780X_{1}X_{5}-0.1083X_{1}X_{6}-\\ 0.0111X_{2}X_{3}+0.0817X_{2}X_{4}+0.0405X_{2}X_{5}-0.0402X_{2}X_{6}-0.2873X_{3}X_{4}-\\ 0.1011X_{3}X_{5}-0.1967X_{3}X_{6}-0.0283X_{4}X_{5}+0.2186X_{4}X_{6}-0.0289X_{5}X_{6}-\\ 0.769X_{1}^{2}-0.2811X_{2}^{2}-1.64X_{3}^{2}-1.13X_{4}^{2}-0.2342X_{5}^{2}-0.0627X_{6}^{2}\# \end{array}$$

The ANOVA results (Table 2) indicated that the model was highly significant (*F* = 16.76, *P* < 0.0001) with a non-significant lack of fit (*P* = 0.2443), confirming model adequacy. The *R*^2^ value of 0.8864 indicated that 88.64% of the yield variance was explained by the model. Ultrasonic time (*X_3_*) and liquid–solid ratio (*X_4_*) were the most significant factors (*P* < 0.0001), followed by soaking time (*X_1_*) and ultrasonic temperature (*X_6_*) (*P* < 0.01) and ultrasonic power (*X_5_*) (*P* < 0.05). NaOH concentration (*X_2_*) was not significant (*P* > 0.05). The influence ranking was *X_3_* > *X_4_* > *X_1_* > *X_6_* > *X_5_* > *X_2_*. Significant two-factor interactions were observed for *X_3_X_4_* (*P* < 0.01) and for *X_3_X_6_* and *X_4_X_6_* (*P* < 0.05). Quadratic terms *X_3_*2 and *X_4_*2 were highly significant (*P* < 0.0001), and *X_1_*2 was also significant (*P* < 0.001), confirming nonlinear effects.

**Table 2:** ANOVA of the RSM model.

**Source**	**Sum of squares**	**Degree**	**Mean square**	***F*-value**	***P*-value**
Model	267.72	27	9.92	16.76	<0.0001**
A	8.25	1	8.25	13.95	0.0004**
B	1.22	1	1.22	2.06	0.0543
C	17.30	1	17.30	29.03	<0.0001**
D	12.66	1	12.66	21.20	<0.0001**
E	2.98	1	2.98	4.99	0.0286*
F	4.50	1	4.50	7.54	0.0078**
AB	0.5023	1	0.5023	0.84	0.3607
AC	0.0029	1	0.0029	0.005	0.9445
AD	0.0356	1	0.0356	0.06	0.8070
AE	0.3891	1	0.3891	0.65	0.4208
AF	0.7504	1	0.7504	1.27	0.2648
BC	0.0079	1	0.0079	0.0133	0.9086
BD	0.4274	1	0.4274	0.71	0.3989
BE	0.1048	1	0.1048	0.1771	0.6754
BF	0.1032	1	0.1032	0.1744	0.6778
CD	5.28	1	5.28	8.93	0.0041**
CE	0.6541	1	0.6541	1.11	0.2975
CF	2.48	1	2.48	4.20	0.0453*
DE	0.0512	1	0.0512	0.0865	0.7697
DF	3.06	1	3.06	5.17	0.0267*
EF	0.0535	1	0.0535	0.0904	0.7648
A^2^	8.21	1	8.21	13.88	0.0004**
B^2^	1.10	1	1.10	1.85	0.1785
C^2^	37.47	1	37.47	63.31	<0.0001*
D^2^	17.74	1	17.74	29.98	<0.0001*
E^2^	0.7614	1	0.7614	1.29	0.2613
F^2^	0.0546	1	0.0546	0.0923	0.7624
Residual	34.32	58	0.5918		
Lack of Fit	30.70	49	0.6266	1.56	0.2443
Pure Error	3.62	9	0.4023		
Cor Total	302.05	85			
R^2^	0.8864				
Adjusted R^2^	0.8335				

** indicates highly significant (*p* < 0.01).

* indicates significant (*p* < 0.05).

#### 3.1.3 Visualization and interpretation of RSM

**Fig. 3:**
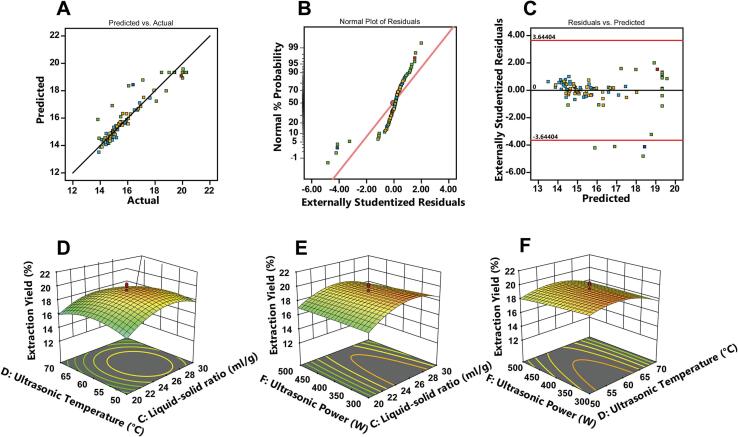
Diagnostic plots confirming model adequacy: (A) predicted versus actual values, (B) normal probability plot of residuals, and (C) studentized residuals versus predicted values. (D–F) Three-dimensional response surface plots showing interaction effects between selected variable pairs on CRP extraction yield.

Three-dimensional response surface and contour plots (Fig. 3A–F) were generated to visualize pairwise interactions while holding the remaining variables at their central levels. Steeper response surfaces and elliptical contour patterns indicated stronger interactions. Ultrasonic time and liquid–solid ratio showed the strongest interactive effect, followed by ultrasonic time and temperature and by liquid–solid ratio and temperature. These interactions displayed steep gradients and narrow ellipses, indicating strong synergy among cavitation intensity, solvent penetration, and polysaccharide solubilization. In contrast, ultrasound power and NaOH concentration exhibited weaker effects within the investigated ranges.

#### 3.1.4 Determination and validation of RSM-optimal conditions

Numerical optimization produced theoretical optimal conditions of ultrasonic time 35.73 min, soaking time 140.82 min, NaOH concentration 0.544 mol/L, liquid–solid ratio 26.09 mL/g, ultrasonic power 300 W, and ultrasonic temperature 56.99 °C. These values were rounded to 36 min, 141 min, 0.54 mol/L, 26 mL/g, 300 W, and 57 °C for practical implementation. Under these conditions, the experimental yield reached 19.89%, which agreed with the predicted value within the 95% confidence interval. Because RSM is limited by its quadratic structure in capturing complex nonlinear relationships and global optima, SVR was subsequently applied for comparative evaluation.

### 3.2 Machine learning-based optimization using SVR

#### 3.2.1 Model performance evaluation and diagnostic validation

**Fig. 4:**
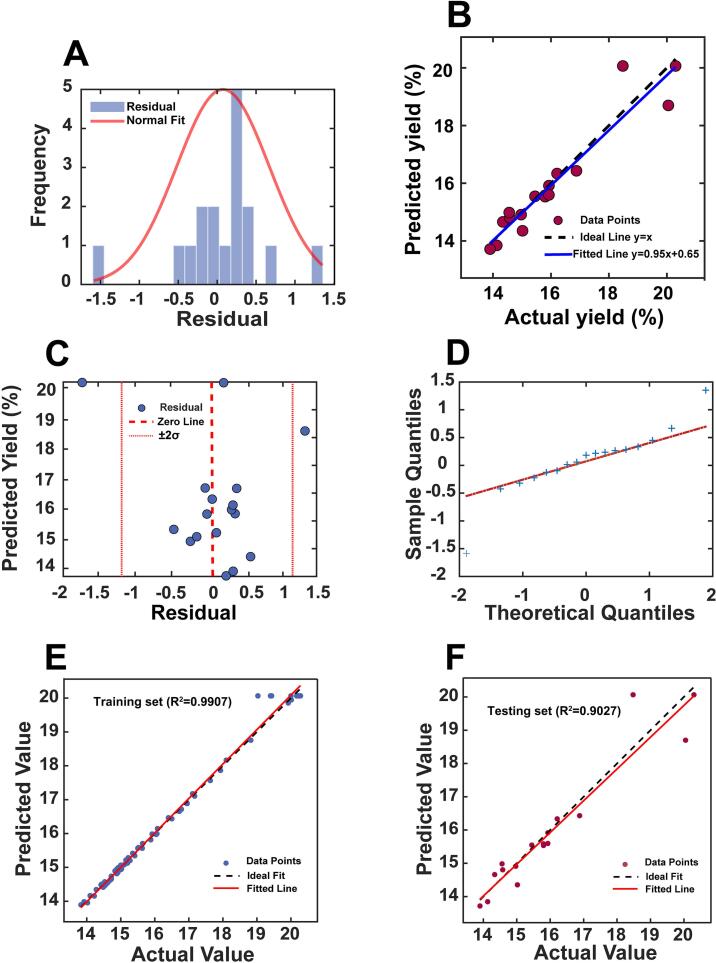
Diagnostic plots of the SVR model for CRP yield prediction: (A) histogram of standardized residuals with normal fit; (B) predicted versus actual yields with fitted and 1:1 reference line; (C) residuals versus fitted values with ± 2*σ* limits; (D) normal Q–Q plot of residuals; (E) parity plot of the training set; and (F) parity plot of the testing set.

The SVR model demonstrated strong predictive performance for both training and testing datasets. *R*^2^ values were 0.9907 for training and 0.9027 for testing, indicating that more than 90% of the variance in unseen data was explained. Testing errors were RMSE = 0.5813%, MAE = 0.4021%, and MAPE = 2.39%, all within acceptable bioprocess prediction limits. The generalization gap (Δ*R*^2^ = 0.0880) remained below 0.10, confirming stable performance after nested five-fold cross-validation with optimal hyperparameters *C* = 10.0, *γ* = 1.0, *ε* = 0.01, yielding a mean cross-validation RMSE = 0.1383 ± 0.0362) [32]. Residual diagnostics (Fig. 4A–D) confirmed model adequacy. Residuals were normally distributed (Lilliefors p = 0.324) and centered near zero. Predicted and observed values closely followed the 1:1 reference line. No serial correlation was detected (Durbin–Watson = 1.599). Mild heteroscedasticity at extreme yield values (Breusch–Pagan *P* = 0.0042) was attributable to two observations located near factorial boundaries with higher experimental variability. Nevertheless, 95.3% of residuals fell within ± 1σ, supporting prediction reliability. Two testing outliers (actual yields 20.1% and 20.6%) were located at sparsely sampled boundaries, where only three training points exceeded 20%. The final model contained 63 support vectors (≈91% of the training set), reflecting process complexity. The relatively narrow RBF kernel (γ = 1.0) and small ε = 0.01 enabled accurate modeling of localized nonlinear behavior without evidence of overfitting, as indicated by stable error metrics and a low Δ*R*^2^.

#### 3.2.2 Comparative assessment of SVR versus RSM

Direct comparison with RSM showed that SVR achieved consistently higher predictive accuracy. The testing *R*^2^ increased from 0.8751 to 0.9027 (+3.2%), and RMSE decreased from 0.6585% to 0.5813% (−11.7%). Although SVR showed a larger Δ*R*^2^ (0.088 vs. 0.0403), this reflects underfitting in the RSM model rather than overfitting in SVR. A paired *t*-test (*n* = 17) indicated no significant difference in mean prediction errors (*P* = 0.868), likely due to the limited size of the test set. Nevertheless, SVR improved prediction accuracy in high-yield regions (>17–19%). Visual comparisons in Fig. S1 further support these findings, showing that the SVR model yields narrower absolute error ranges, more compact residual distributions, and improved tail behavior. SVR exhibits substantially enhanced predictive accuracy and error stability. The median absolute error is reduced by 55.2%, decreasing from 0.87% to 0.39%, together with a 48.4% reduction in the interquartile range, indicating improved robustness against dispersion. Across the full yield range, MAE values remain consistently lower, with the most pronounced improvement observed at high-yield intervals (0.96% vs. 1.87%). In addition, the 90th-percentile error decreases from 1.89% to 0.68%, and residual analysis confirms a more symmetric error distribution with reduced variance.

Single-variable analyses further revealed threshold effects and asymmetric responses that are not adequately described by quadratic polynomials. NaOH concentration exhibited an optimum at 0.50–0.55 mol/L, with a sharp yield decrease below 0.45 mol/L due to insufficient cell wall swelling and a gradual decline above 0.60 mol/L caused by progressive β-elimination. Ultrasonic time showed diminishing returns beyond 40 min at suboptimal liquid–solid ratios but continued to increase yield up to 60 min under optimal conditions. These multi-inflection and context-dependent responses explain why RSM underestimated yield in high-performance regions and overestimated yield in low-performance regions, whereas SVR provided balanced predictions.

These findings demonstrate that SVR captures nonlinear behavior in the UAAE process, which involves strong interactions, threshold effects, and asymmetric response surfaces that violate the assumptions of quadratic RSM. The improved SVR performance reflects its ability to represent such complexity through kernel-based learning rather than predefined polynomial constraints.

#### 3.2.3 Variable sensitivity and mechanistic factor importance

The nonlinear interactions observed above prompted evaluation of variable influence on UAAE performance. SVR-derived importance was quantified using permutation importance, support vector contribution, and partial dependence analysis [33]. Integrated results are summarized in Table S2, and marginal effect profiles are presented in Fig. S2. Ultrasonic time was the most influential variable, followed by liquid–solid ratio and soaking time. NaOH concentration showed moderate importance, while ultrasonic power and temperature exhibited lower influence within the tested ranges. Partial dependence plots provided further insight into marginal factor effects (Fig. S2). Soaking time showed an asymmetric optimum at 134.2 min, with yield increasing rapidly to the optimum and declining thereafter, indicating polysaccharide degradation or impurity re-dissolution under excessive soaking. NaOH concentration exhibited a narrow optimum at 0.5 mol/L, with steep yield losses outside 0.45–0.55 mol/L, confirming threshold-type behavior. The liquid–solid ratio presented a broad optimum at 26.9 mL/g, beyond which dilution likely reduced cavitation efficiency. Ultrasonic temperature showed a mild optimum at 57.3 °C with stable yields across 50–65 °C, indicating a secondary role. Ultrasonic time displayed a sharp optimum at 31.4 min, with prolonged exposure inducing degradation. Ultrasonic power peaked at 379.4 W, with insufficient cavitation at lower power and structural damage at higher power. Small deviations between predicted and experimental optima confirm the high predictive accuracy of the SVR model and its ability to capture nonlinear process behavior beyond polynomial RSM.

This resulting hierarchy indicates that ultrasonic time and liquid–solid ratio function as primary control variables governing cavitation intensity and mass transfer, soaking time acts as a key pre-treatment parameter facilitating NaOH penetration, NaOH concentration and ultrasonic power serve as secondary modulators fine-tuning extraction efficiency, and temperature exerts tertiary influence within the tested range, demonstrating that mechanical–hydraulic drivers dominate UAAE performance.

#### 3.2.4 Global optimization and experimental validation of SVR

The optimized SVR model was experimentally validated. The observed maximum yield of 20.30% closely matched the predicted value 20.06%, with a relative error of 1.16%, confirming model accuracy. Compared with RSM (predicted 19.89%, observed 18.26%), SVR showed superior agreement between predicted and observed yields in high-performance regions and reduced RMSE substantially. Temporal yield tracking (Fig. S3A–B) demonstrated close agreement between SVR predictions and experimental trajectories. Under SVR-optimal conditions, triplicate validation yielded relative deviations below 1%, confirming robustness and reproducibility. The final SVR-optimal parameters were ultrasonic time 35.05 min, soaking time 137.86 min, NaOH concentration 0.55 mol/L, liquid–solid ratio 25.90 mL/g, ultrasonic power 325.40 W, and ultrasonic temperature 56.35 °C. For practical implementation, these values were rounded to 35 min, 138 min, 0.55 mol/L, 26 mL/g, 325 W, and 56.5 °C. Under these conditions, the measured CRP yield reached 20.09 ± 0.10%, demonstrating high predictive accuracy and practical feasibility of the SVR model.

#### 3.2.5 Selection of final industrial extraction conditions

Table 3 summarizes the CCD center conditions, the RSM- and SVR-derived optima, experimental validation results, and the final recommended industrial conditions, together with the corresponding polysaccharide yield and energy performance normalized per kilogram of raw material. Both optimization models predicted similar optimal parameter regions, and experimental validation confirmed comparable extraction yields. The SVR-derived optimum exhibited a slightly higher predicted yield than the RSM-derived optimum, while requiring nearly identical energy input. The SVR model provided a balanced parameter combination, including marginally lower ultrasonic temperature and shorter effective processing duration, which is advantageous for preserving polysaccharide structural integrity and improving operational stability. For industrial feasibility, final recommended extraction conditions were derived from the SVR-based optimum with minor parameter rounding to facilitate process control, scalability, and reproducibility without compromising extraction performance or energy intensity. Under these conditions, the experimentally validated yield and energy consumption remained consistent with SVR predictions. The SVR-optimized conditions were therefore selected as the final recommended extraction process, as they provide an effective balance between extraction performance, predictive reliability, operational robustness, and scalability for industrial application.

**Table 3:** Comparison of process parameters, extraction performance, and energy metrics among CCD center conditions, RSM, SVR, and industrial conditions.

Parameters	CAE (Control)^a^	CCD Center	RSM Optimum^b^	SVR Optimum	Experimental Validation c	Industrial Recommendation^d^
*Process Parameters*						
Soaking time (min)	140	120	140.82	137.86	138	138
NaOH concentration (mol/L)	0.54	0.50	0.54	0.55	0.55	0.55
Liquid–solid ratio (mL/g)	25	25	26.09	25.9	26	26
Temperature (°C)	100	60.0	56.99	56.35	56.5	56.5
Ultrasonic time (min)	—	30.00	35.73	35.05	35.00	35.00
Ultrasonic power (W)	—	400	300	325.4	325	325
*Performance Metrics*						
Polysaccharide yield (%)	13.07 ± 0.35	19.24 ± 0.08	19.89	21.00	20.09 ± 0.10	20.09
Improvement (%) e		47.2	52.2	60.7	53.7	53.7
Total process time (min)	260	150	176.55	172.91	173	173
Production rate (%/h)	3.02	7.70	6.76	7.29	6.97	6.97
SEC (kWh/kg) f	918.13	51.98	46.87	46.89	49.07	49.07
CO_2_ emissions (kg/kg)	734.51	41.58	37.49	37.51	39.26	39.26

a CAE extraction: 100 °C for 2 h in a thermostatic water bath (1200 W).

b RSM-derived optimal conditions predicted by the model.

c Experimental validation of the SVR-derived optimal conditions (mean ± SD, n = 3).

d Rounded parameter set for industrial feasibility, with predicted performance metrics based on the experimental validation of the SVR-derived optimum.

e Improvement calculated as [(Yield − Yield of CAE)/Yield of CAE] × 100%.

f SEC was calculated as energy consumed only during the extraction stage (ultrasonication and/or heating) per kg of raw material; the soaking step was considered energy-neutral and thus excluded.

### 3.3 Economic analysis of different extraction methods

After establishing an optimized UAAE process through SVR-based modeling, a techno-economic assessment was conducted to evaluate its industrial feasibility relative to CAE. As summarized in Table 3, UAAE achieved an extraction yield of 20.09 ± 0.10%, representing a 53.7% improvement over CAE. Extraction time decreased from 120 min to 35 min, corresponding to a 70.8% reduction and a marked increase in production rate. Specific energy consumption declined to 49.07 kWh/kg polysaccharide compared with 918.13 kWh/kg for CAE, resulting in a proportional reduction in CO_2_ emissions. The carbon intensity of UAAE falls below ranges typically reported for plant-based bioproducts, whereas CAE exhibits a substantially higher footprint, confirming that UAAE provides an efficient and low-carbon extraction route. These environmental and economic advantages were achieved concurrently with improved polysaccharide quality. UAAE-derived CRP exhibited superior preservation of high-molecular-weight fractions, enhanced maintenance of helical conformations confirmed by Congo red, TGA, and XRD analyses, and stronger antioxidant activity. This concurrent improvement in sustainability and bioactivity arises from ultrasonic cavitation-driven mechanical disruption of the cell wall while minimizing thermal and chemical degradation through reduced extraction time and moderate operating temperature. From an industrial perspective, this dual advantage extends beyond cost reduction to value enhancement, as high-molecular-weight bioactive polysaccharides command higher market value in functional food and nutraceutical applications. Combined with improved yield and markedly reduced emissions, UAAE offers integrated techno-economic and environmental advantages, including lower operating costs, reduced regulatory burden, and higher product value, positioning UAAE as a commercially viable and environmentally responsible process for large-scale polysaccharide production aligned with circular bioeconomy and sustainable ingredient development.

### 3.4 Mechanistic Insights into UAAE via Microstructural analysis

SEM, XRD, FT-IR, and TGA were used to investigate microstructural and compositional changes in the cell walls of *Chroogomphus rutilus* before and after extraction. These analyses provide mechanistic insight into how UAAE disrupts cell wall integrity and promotes polysaccharide release.

#### 3.4.1 SEM

**Fig. 5:**
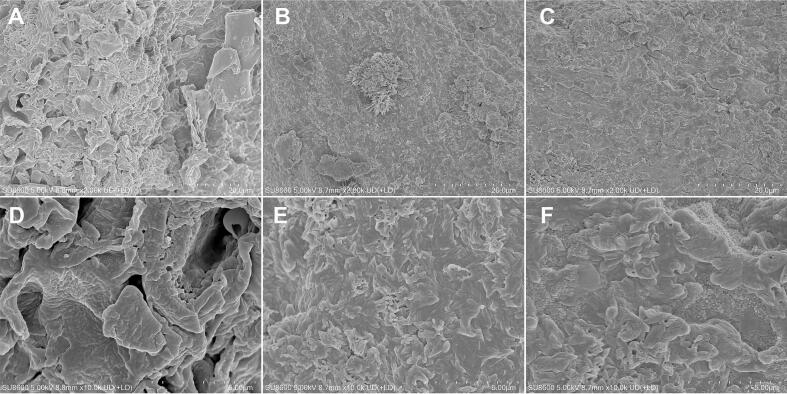
SEM micrographs of *Chroogomphus rutilus* raw material and extraction residues: (A, D) CR; (B, E) CR-UAAE; (C, F) CR-CAE. Magnifications: (A–C) × 2,000; (D–F) × 10,000.

Microstructural changes induced by different extraction processes were examined by SEM to clarify the mechanisms underlying differences in extraction efficiency (Fig. 5). Untreated mushroom powder (CR) exhibited a well-organized cell wall architecture characterized by densely packed fibrillar networks and smooth lamellar arrangements. At low magnification (×2.00 k, Fig. 5A), the cell walls formed a compact three-dimensional network of interwoven sheet-like fragments, indicating intact structural integrity. At higher magnification (×10.0 k, Fig. 5D), the walls showed uniform thickness and clearly defined stratified layers, consistent with the chitin–glucan complex that constitutes the fungal cell wall matrix. In contrast, residues obtained after UAAE (CR-UAAE) exhibited pronounced structural disruption. At × 2.00 k magnification (Fig. 5B), the previously compact network was substantially loosened, with numerous voids and fissures across the surface. High-magnification images (Fig. 5E) revealed irregular surface roughening, marked delamination of lamellar layers, multiple cavities and depressions, and fragmented edges with nonuniform rupture patterns. By comparison, residues from CAE (CR-CAE) showed intermediate morphology. At × 2.00 k magnification (Fig. 5C), partial swelling and moderate network disruption were observed, but the overall framework remained recognizable. At higher magnification (Fig. 5F), surface roughening and partial delamination were evident, although substantial portions of intact lamellar structures persisted, indicating incomplete wall disintegration and limited alkali penetration. These observations demonstrate that the superior efficiency of UAAE arises from the combined action of ultrasonic cavitation and alkaline hydrolysis. Cavitation generates intense mechanical forces—such as microjets and shockwaves—that physically disrupt the alkali-softened wall matrix. Simultaneously, alkaline conditions solubilize lignin and hemicellulose, reducing wall rigidity and preventing reaggregation of wall polymers [17], [30]. This dual mechanism enables extensive and irreversible cell wall breakdown and significantly enhances extraction efficiency relative to chemical treatment alone.

#### 3.4.2 XRD

**Fig. 6:**
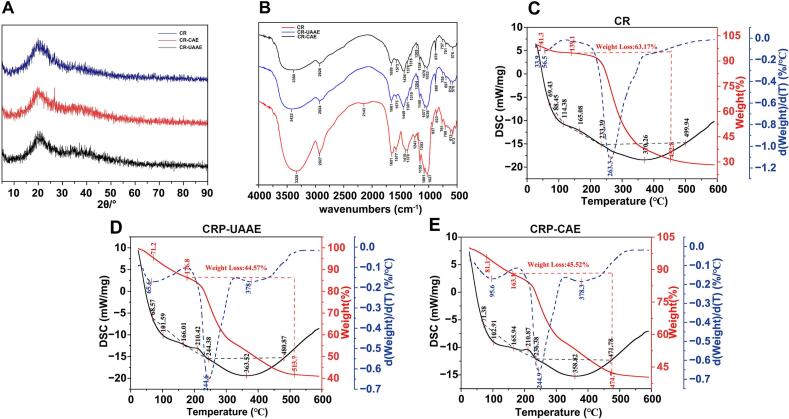
Characterization of CR, CR-UAAE, and CR-CAE by (A) FT-IR, (B) XRD, and (C–E) TGA/DTG analyses showing thermal degradation and mass-loss behavior.

XRD patterns of native CR and residues after UAAE and CAE are shown in Fig. 6A to assess extraction-induced changes in the crystalline organization of cell wall components. Native CR exhibited a prominent diffraction peak at 19.9° (2θ) with a maximum intensity of 117.2 counts, assigned to the (200) plane of cellulose I and indicating a semicrystalline matrix comprising of crystalline and amorphous regions [38]. After CAE, the main peak shifted slightly to 19.5° (2θ) with reduced intensity (106.2 counts), corresponding to a 9.4% decrease in crystallinity, consistent with lattice swelling and partial structural disruption. In contrast, UAAE residues retained the peak position at 19.9° (2θ) but showed a further decrease in intensity (104.3 counts), indicating an 11.0% reduction in crystallinity without detectable lattice displacement. This pattern indicates disruption of crystalline domains while preserving crystallographic alignment. All samples exhibited a broad shoulder near 22° (2θ), characteristic of native cellulose. In the 33°–42° (2θ) region, CR-UAAE showed slightly elevated and more diffuse background intensity relative to CR and CR-CAE, suggesting ultrasound-induced rearrangement of semi-ordered domains or reorganization of residual wall polymers. These features are consistent with localized mechanical stress and transient heating during cavitation collapse. Both extraction methods reduced crystallinity; however, UAAE modulated the cellulose matrix more selectively. Preservation of the main diffraction peak with partial intensity loss suggests preferential disruption of amorphous regions with limited reorganization of crystalline domains. Such targeted deconstruction increases porosity and solvent accessibility while maintaining polysaccharide integrity [34]. These results provide mechanistic evidence that UAAE enhances extraction efficiency through controlled mechanical–chemical modulation that balances disruption with structural preservation.

#### 3.4.3 FT-IR

FT-IR spectra of CR, CR-UAAE, and CR-CAE (Fig. 6B) revealed extraction-dependent changes in bonding environments associated with lignin, cellulose, and hemicellulose. In the aromatic region (1400–1600 cm⁻^1^), native CR showed characteristic lignin bands at 1651, 1595, and 1435 cm⁻^1^. After UAAE, these bands shifted to 1673 cm⁻^1^ and the aromatic/aliphatic ratio decreased (I_1595_/I_2927_ = 1.28 vs. 1.45 for CR), indicating cleavage of lignin–carbohydrate linkages [35]. CR-CAE also altered lignin-related bands but introduced a shoulder at 1578 cm^−1^, indicating less selective modification. Cellulose crystallinity, estimated by I_1165_/I_1104_, decreased from 1.12 (CR) to 1.06 (CR-UAAE) and 0.98 (CR-CAE), indicating progressive decrystallization. The hemicellulose-associated carbonyl band at 1735 cm^−1^ decreased markedly, with I_1735_/I_1040_ declining to 0.31 (CR-UAAE) and 0.28 (CR-CAE), confirming effective hemicellulose removal [36]. O–H stretching vibrations (3200–3600 cm⁻^1^) shifted from 3395 cm⁻^1^ (CR) to 3424 cm⁻^1^ (CR-UAAE) and 3325 cm⁻^1^ (CR-CAE), accompanied by reduced I_3400_/I_2927_ ratios, indicating weakening of the hydrogen-bond networks. In the fingerprint region, attenuation of C–O and C–C stretching bands (1000–1200 cm⁻^1^ and ∼ 1320 cm⁻^1^) was observed, with I_1040_/I_2927_ decreasing from 2.76 (CR) to 2.31 (CR-UAAE) and 1.89 (CR-CAE), consistent with cleavage of glycosidic and backbone bonds. UAAE preferentially weakened lignin-associated signals and hydrogen bonding while maintaining comparatively higher glycosidic integrity, as indicated by the higher I_1165_/I_1040_ ratio (1.06 vs. 0.98 for CAE). In contrast, CAE produced broader attenuation across C–O and C–C vibrations, suggesting more extensive polysaccharide degradation. These spectral features indicate that UAAE enhances mass transfer and polysaccharide release while better preserving molecular integrity.

#### 3.4.4 TGA

TGA profiles of CR, CR-UAAE, and CR-CAE are shown in Fig. 6C–E. All samples exhibited four major mass-loss stages. Native CR decomposed at *T*_1_ = 69.43 °C, *T*_2_ = 114.38 °C, *T_3_* = 233.39 °C, and *T_4_* = 499.94 °C. Both extraction residues showed a pronounced increase in *T_2_* to approximately 165 °C, consistent with removal of thermally labile fractions. CR-UAAE exhibited the highest *T_3_* (244.38 °C), exceeding that of CR-CAE (236.38 °C), indicating superior retention of thermally stable components. The initial dehydration enthalpy (Δ*H*_12_) increased markedly after extraction, reaching 690.51 J/g for CR-UAAE and 589.17 J/g for CR-CAE, corresponding to 4.3- and 3.7-fold increases relative to CR (159.09 J/g). This increase reflects enhanced water-binding capacity associated with cavitation-induced structural modification. During oxidative decomposition (Δ*H*_23_), all samples released comparable heat. The final decomposition enthalpy (Δ*H*_34_) of CR-UAAE (3586.1 J/g) slightly exceeded that of native CR (3559.0 J/g), whereas CR-CAE decreased to 2609.1 J/g. These results indicate that UAAE preserves or enriches energy-dense components, while CAE removes a greater fraction of combustible material. The elevated Δ*H*_12_ and sustained Δ*H*_34_ of CR-UAAE are consistent with strengthened water–matrix interactions and partial molecular reorganization associated with increased porosity.

#### 3.4.5 Synergistic mechanism of ultrasonic-enhanced extraction

Multi-scale characterization demonstrates that UAAE induces coordinated disruption at cellular, crystalline, and molecular levels. SEM showed conversion of dense cell walls into porous, delaminated networks, exceeding the moderate loosening observed after CAE. XRD revealed an 11% reduction in cellulose crystallinity after UAAE while retaining the 19.9° (2θ) peak, indicating selective disruption of amorphous domains with preserved crystallographic alignment. FT-IR confirmed weakening of lignin–carbohydrate associations and hydrogen-bond interactions, while glycosidic linkages remained comparatively intact relative to CAE. TGA further indicated higher dehydration enthalpy and retained final energy content for CR-UAAE than for CR-CAE, consistent with enhanced water–matrix interactions and partial molecular reorganization. This synergy arises from complementary effects of ultrasonic cavitation and alkaline hydrolysis. Cavitation generates microjets, shockwaves, and shear forces that fragment and delaminate the alkali-softened matrix, enhancing solvent penetration and diffusion. Concurrent alkaline hydrolysis selectively solubilizes hemicellulose- and lignin-associated components, loosening the matrix while limiting uncontrolled depolymerization. The ultrasonic field also suppresses polymer reaggregation, facilitating release of comparatively intact polysaccharides, consistent with the bond integrity and thermal stability indices [36], [37]. This mechanism follows a coordinated pathway in which alkali pretreatment weakens hydrogen bonding and partially removes matrix-associated components, cavitation-driven fragmentation increases porosity and exposes less ordered domains while maintaining peak alignment, and enhanced mass transfer promotes polysaccharide solubilization with limited chain degradation. These coordinated effects account for the improved efficiency, selectivity, and structural preservation achieved by UAAE.

### 3.5 Physicochemical properties of CRP-UAAE and CRP-CAE

#### 3.5.1 Chemical composition

The chemical compositions of CRPs obtained by different extraction methods are summarized in Table 4. The total carbohydrate content of CRP-UAAE (66.59 ± 1.67%) was significantly higher than that of CRP-CAE (58.33 ± 2.82, *P* < 0.05), which can be attributed to ultrasonic cavitation generating localized microenvironments that disrupt cross-linked polysaccharide networks within the fungal cell wall and thereby enhance solvent accessibility to embedded carbohydrate fractions [38]. In addition, mechanical shear forces and transient thermal gradients produced during cavitation collapse may further weaken the associations between hemicellulose- and lignin-linked components, facilitating polysaccharide release [38], [39]. The protein content of CRP-UAAE (3.06 ± 0.06%) was also significantly higher than that of CRP-CAE (1.27 ± 0.07%, *P* < 0.05), suggesting that ultrasonic treatment promotes the co-extraction of proteinaceous components, as a result of more extensive cell wall disruption and enhanced mass transfer under cavitation conditions. Previous studies have demonstrated that ultrasound improves the solubilization of intracellular or wall-associated proteins by mechanically breaking structural barriers and exposing protein–polysaccharide complexes [40]. The higher protein content in CRP-UAAE therefore reflects the broader extraction capability of ultrasound rather than selective protein enrichment. UAAE yielded a slightly higher uronic acid content (0.78 ± 0.01%) than CAE (0.76 ± 0.06%), although this difference was not statistically significant (*P* > 0.05). This result indicates that both extraction methods exhibit comparable efficiency in recovering uronic acid–containing polysaccharides. The marginal increase observed for UAAE may be associated with improved solvent penetration and mechanical disruption under ultrasonic conditions, which facilitate the release of acidic polysaccharide domains associated with the cell wall matrix. Similar trends have been reported in previous studies showing that ultrasound enhances solvent accessibility without necessarily inducing substantial compositional shifts [41].

**Table 4:** Chemical composition and physicochemical properties of CRP obtained by UAAE and CAE.

**Parameter**	**UAAE**	**CAE**
*Chemical composition*		
Total carbohydrates (%)	66.59 ± 1.67^a^	58.33 ± 2.82^b^
Uronic acid (%)	0.78 ± 0.01^a^	0.76 ± 0.06^a^
Protein (%)	3.06 ± 0.06^a^	1.27 ± 0.07^b^
*Monosaccharide composition (mol, %)*		
Mannose (Man)	4.46 ± 0.07^a^	4.81 ± 0.01^a^
Rhamnose (Rha)	0.11 ± 0.04^a^	0.08 ± 0.03^a^
Glucuronic acid (GlcA)	0.12 ± 0.01^a^	0.11 ± 0.005^a^
Glucose (Glc)	83.35 ± 0.05^b^	86.35 ± 0.13^a^
Galactose (Gal)	7.65 ± 0.11^a^	6.26 ± 0.13^b^
Xylose (Xyl)	1.11 ± 0.06^a^	1.40 ± 0.08^a^
Fucose (Fuc)	1.19 ± 0.04^a^	1.00 ± 0.09^a^
*Physicochemical properties*		
Conductivity (mS/cm)	0.17 ± 0.003^b^	0.38 ± 0.001^a^
Viscosity (mPa·s)	0.85 ± 0.03^b^	2.56 ± 0.06^a^
Zeta potential (mV)	−13.7 ± 0.20^b^	−15.2 ± 0.08^a^
Particle size (nm)	224.7 ± 4.50^b^	373.3 ± 2.00^a^

**Note:** Data are presented as mean ± SD (n = 3). Different superscript letters within the same row indicate significant differences according to Tukey’s test (*P* < 0.05).

#### 3.5.2 Monosaccharide composition

**Fig. 7:**
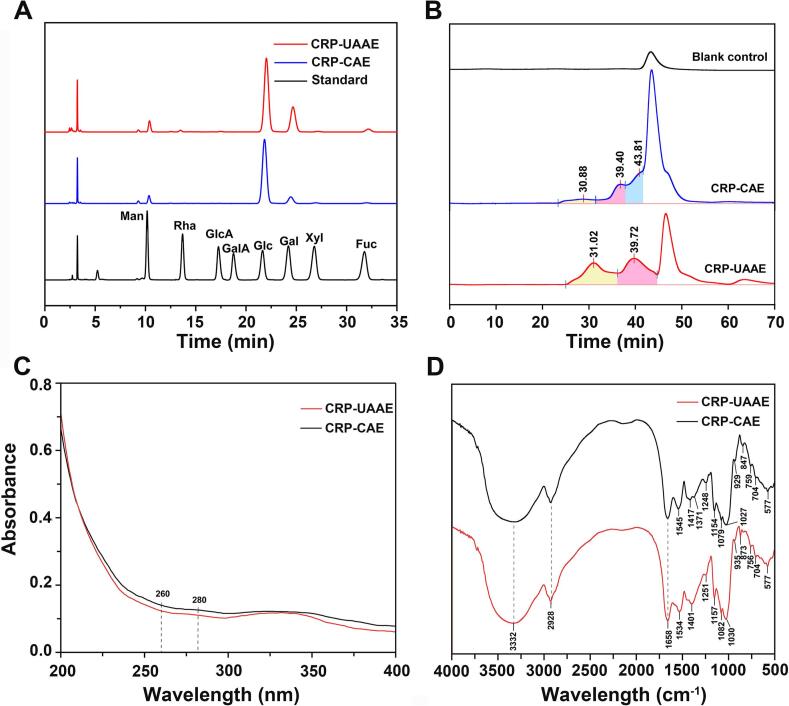
Characterization of CRP polysaccharides: (A) HPLC chromatograms of monosaccharide composition, (B) HPGPC molecular weight distribution, (C) UV–Vis absorption spectra, and (D) FT-IR spectra of functional groups.

The monosaccharide compositions of CRPs obtained by UAAE and CAE are shown in Fig. 7A. HPLC analysis identified eight monosaccharides, Man, Rha, GlcA, GalA, Glc, Gal, Xyl, and Fuc, with Glc as the dominant component (85.35% for UAAE and 86.35% for CAE) in both samples, indicating that the extracted polysaccharides are primarily glucose-based polymers. Although the overall profiles were similar, relative molar proportions differed between methods. CRP-UAAE contained slightly less Glc and more Gal than CRP-CAE, while Man content remained comparable (4.46% vs. 4.80%), Minor sugars, including Fuc, Xyl, and Rha, were modestly enriched in CRP-UAAE relative to CRP-CAE, indicating improved release of residues that are more deeply embedded or tightly associated within the wall matrix. These differences likely reflect the combined action of ultrasonic cavitation and alkaline swelling, where cavitation-induced shear, microjets, and localized microenvironments disrupt dense polysaccharide networks, while alkaline conditions weaken hydrogen bonding and enhance wall permeability [30], [41]. The predominance of Glc suggests glucan-type structures, whereas the presence of Gal, Rha, and uronic acids indicates co-extraction of structurally diverse fractions, contributing to a heterogeneous polysaccharide mixture.

#### 3.5.3 Molecular weight distribution

The molecular weight distributions of CRPs extracted by UAAE and CAE were determined by HPGPC (Fig. 7B). CRP-UAAE exhibited two dominant fractions at retention times of 31.02 min and 39.72 min, corresponding to molecular weights of 7.54 × 10^5^ Da and 1.32 × 10^4^ Da, which accounted for 45.68% and 54.32% of the total peak area, respectively. In contrast, CRP-CAE showed three peaks at 30.88 min, 39.40 min, and 43.81 min, with a trimodal distribution dominated by low-molecular-weight species at 1.43 × 10^3^ Da (51.11%), accompanied by a medium- molecular-weight component at 1.54 × 10^4^ Da (35.18%) and only a minor high- molecular-weight fraction at 8.08 × 10^5^ Da (13.70%). Based on peak-area-weighted calculation, the apparent weight-average molecular weight of CRP-UAAE was approximately 3.5 × 10^5^ Da, about three times higher than that of CRP-CAE (1.2 × 10^5^ Da), indicating that UAAE better preserves high-molecular-weight polysaccharide chains. These distributions reflect distinct extraction mechanisms. Ultrasonic cavitation in UAAE enhances cell wall disruption and mass transfer while limiting prolonged chemical exposure, whereas extended alkaline treatment during CAE promotes β-elimination and glycosidic bond cleavage, yielding smaller fragments [9], [41]. Enrichment of high- and medium-molecular-weight fractions in UAAE-derived CRP is functionally advantageous, as polysaccharides within the 100–1000 kDa range are frequently associated with enhanced bioactivities [6].

#### 3.5.4 UV–Vis spectroscopy

UV–Vis spectroscopy was used to assess the purity of CRPs obtained by UAAE and CAE, focusing on residual protein and nucleic acid contamination. As shown in Fig. 7C, both CRP-UAAE and CRP-CAE exhibited characteristic polysaccharide absorption at 200–220 nm, followed by a rapid decline at higher wavelengths. Weak absorption bands at 260 nm and 280 nm, attributable to nucleic acids and proteins, respectively, were detected in both samples; however, signal intensities differed between extraction methods. CRP-CAE showed higher absorbance at 260 nm (*A*_260_ = 0.140) and 280 nm (*A*_280_ = 0.127), whereas CRP-UAAE exhibited lower values indicating reduced co-extraction of nucleic acids and proteins. Although *A*_260_/*A*_280_ ratios were similar for CRP-UAAE and CRP-CAE, the lower absolute absorbance values for CRP-UAAE indicate higher purity. These results suggest that UAAE enhances selectivity for polysaccharides while limiting solubilization of intracellular macromolecules, likely due to reduced extraction time and cavitation-induced protein denaturation and aggregation that facilitate removal during downstream processing.

#### 3.5.5 FT-IR spectroscopy

FT-IR spectra of CRPs obtained by UAAE and CAE (Fig. 7D) displayed typical polysaccharide absorption features with method-dependent differences. Both samples showed strong bands near 3331 cm⁻^1^ attributed to O–H stretching, reflecting extensive intra- and intermolecular hydrogen bonding, and bands near 2926 cm⁻^1^ assigned to C–H stretching of methyl and methylene groups. Clear differences were observed in the 1700–1600 cm⁻^1^ region. CRP-CAE exhibited a stronger band at 1657 cm⁻^1^, attributed to C=O stretching associated with residual protein or uronic acid groups [29]. This band was attenuated in CRP-UAAE, supporting lower protein content, consistent with UV–Vis results. In the fingerprint region (1200–800 cm⁻^1^), both samples showed intense C–O and C–C stretching vibrations characteristic of pyranose rings and glycosidic linkages, with minor shifts indicating subtle differences in sugar composition or backbone conformation. A distinct absorption band at 873 cm⁻^1^, assigned to β-glycosidic linkages, was detected only in CRP-UAAE, suggesting preferential recovery or preservation of β-linked components under ultrasonic conditions [6]. In the 850–500 cm⁻^1^ region, both samples exhibited similar band patterns with minor shifts, consistent with small differences in conformation or substituent environments. These spectral features indicate that UAAE yields CRPs with lower impurity-related signals and preserves extraction-sensitive structural motifs.

#### 3.5.6 SEM

**Fig. 8:**
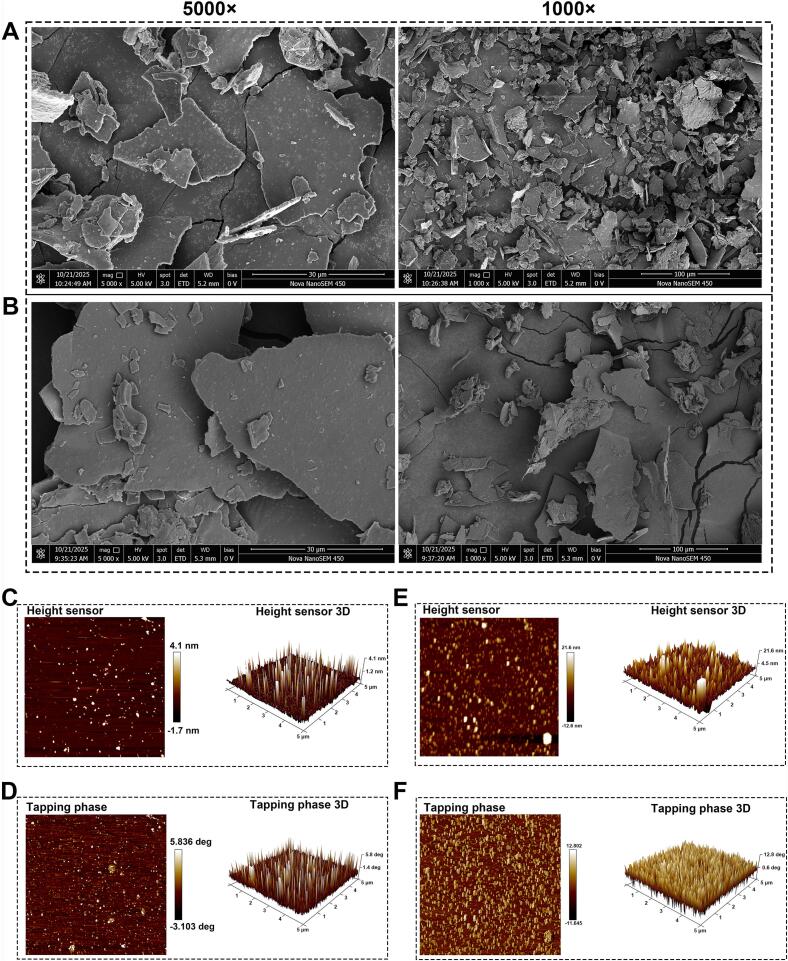
Morphological and topographical characterization of polysaccharides extracted by different methods. (A–B) SEM micrographs of CRP-CAE (A) and CRP-UAAE (B) samples at 1000 × and 5000 × magnifications. (C–F) AFM height and phase images of the polysaccharide surfaces: CRP-CAE (C, D) and CRP-UAAE (E, F).

SEM images revealed pronounced morphological differences between CRPs obtained by CAE and UAAE (Fig. 8A, B). CAE-derived polysaccharides exhibited smooth, plate-like surfaces with dense and compact structures. At 5000 × magnification, particles displayed well-defined edges and minimal porosity, while at 1000 × magnification, large, irregular sheet-like aggregates with heterogeneous size and strong stacking into thick layers were observed. In contrast, UAAE-derived polysaccharides showed markedly rougher surfaces with visible cracks, fissures, and abundant micropores. At 1000 × magnification, particles were smaller, more uniform, and less densely stacked, with open interparticle spacing. UAAE reduced particle size and increased surface roughness relative to CAE. These changes are attributed to acoustic cavitation, in which bubble collapse generates intense shear forces and shockwaves that fragment aggregates, disrupt hydrogen bonding, and suppress reaggregation through microjet impact [42]. The resulting micro- and nanoporous architecture reflects he combined effects of mechanical fracture and sonochemical erosion while retaining the sheet-like morphology characteristic of fungal polysaccharides. Such morphological modifications increase specific surface area, which may enhance solubility, reactivity, and bioavailability, while improved size uniformity favors processability and reproducibility.

#### 3.5.7 AFM

AFM analysis resolved nanoscale morphological differences between CRPs extracted by CAE and UAAE (Fig. 8C–F). Height and phase images revealed differences in particle distribution, structural uniformity, and surface topology. CAE-derived CRPs consisted of sparsely distributed spherical aggregates ranging from submicron to several micrometers, with low height profiles approximately of 1–3 nm and pronounced size heterogeneity. Phase images showed relatively uniform contrast consistent with alkali-induced structural homogenization. In contrast, UAAE-derived CRPs exhibited substantially higher particle density, narrower size distribution, and better-preserved three-dimensional morphology. Particles were predominantly oval or spherical, with height profiles of 2–5 nm. Three-dimensional reconstructions showed continuous surfaces and consistent particle shapes. Quantitative analysis indicated a three- to four-fold increase in particle density and markedly reduced polydispersity for UAAE-derived samples. Phase contrast images showed greater heterogeneity, suggesting coexistence of mechanically distinct microdomains and improved preservation of native conformations features. The increased surface roughness and topographical complexity of CRP-UAAE enhance effective surface area for molecular interactions, supporting superior structural organization, particle uniformity, and morphological integrity relative to CAE, in agreement with compositional and molecular weight analyses.

#### 3.5.8 XRD

**Fig. 9:**
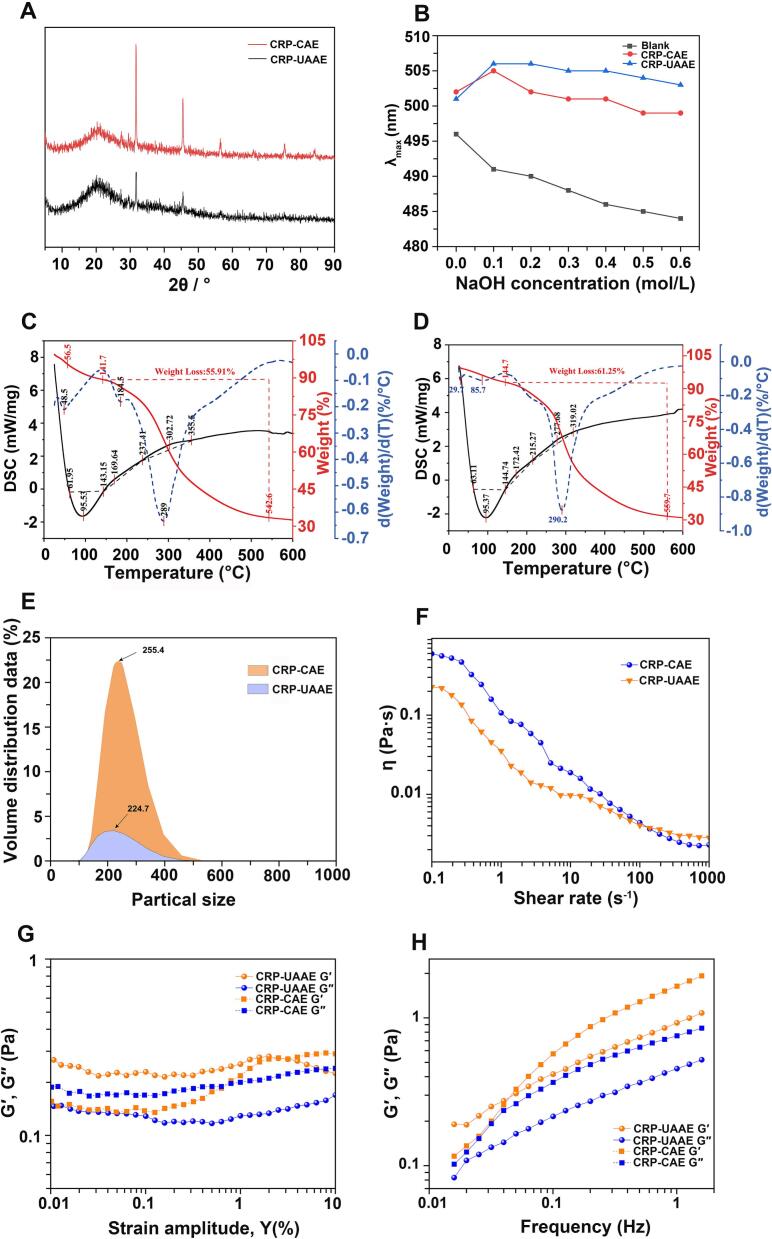
Physicochemical and rheological characterization of CRP-CAE and CRP-UAAE. (A) XRD patterns; (B) Congo red assay showing triple-helix structure; (C–D) DSC/TGA–DTG thermograms; (E) particle size; (F) shear-thinning behavior; (G) strain sweeps of G′ and G″; (H) frequency sweep showing G′ > G″.

XRD analysis was conducted on purified polysaccharides obtained by UAAE and CAE to examine their crystalline organization and the influence of extraction conditions on molecular packing. As shown in Fig. 9A, both samples exhibited a broad diffraction peak centered at approximately 20° (2θ), characteristic of semi-crystalline polysaccharides containing both ordered and amorphous domains. For CRP-UAAE, this peak exhibited an intensity of 114 counts, accompanied by minor secondary peaks at 32° and 45° (2θ) with intensities of 120 and 77 counts, indicating a heterogeneous structure comprising multiple ordered domains. In contrast, CRP-CAE showed a similar primary peak at approximately 20° (2θ) but with lower intensity of about 92 counts and two sharp, intense peaks at 32° and 45° (2θ) with intensities of 266 and 145 counts, respectively, reflecting formation of highly ordered crystalline domains under prolonged alkaline conditions. These differences indicate distinct structural consequences of the two extraction strategies. Extended heating and high alkalinity during CAE promoted molecular chain rearrangement and recrystallization into thermodynamically stable domains. By contrast, the shorter extraction duration and cavitation-induced mechanical during UAAE impeded crystallite growth and favored retention of a more amorphous, potentially native-like organization. This reduced crystallinity is conducive to enhanced hydration and dissolution, while the higher crystallinity of CRP-CAE may increase rigidity at the expense of molecular accessibility. These observations are consistent with molecular weight data showing that UAAE preserves higher-molecular-weight fractions with reduced degradation.

#### 3.5.9 Congo red assay

The presence of helical conformations in polysaccharides extracted by UAAE and CAE was evaluated using the Congo red binding assay. This method is based on the characteristic bathochromic shift in the maximum absorption wavelength (*λ*_max_) that occurs upon specific binding of Congo red to ordered helical structures [6], [11]. As shown in Fig. 9B, the blank control (without NaOH) exhibited a *λ*_max_ of 496 nm, whereas polysaccharides extracted by CAE and UAAE showed red-shifted *λ*_max_ values of 502 nm and 501 nm, respectively. The observed bathochromic shift upon Congo red binding indicates the presence of ordered helical conformations and is consistent with possible triple-helix–like structures. With increasing NaOH concentration (0–0.6 mol/L), the blank sample showed a continuous blue shift, whereas both CAE- and UAAE-derived polysaccharides exhibited an initial increase followed by a gradual decrease in *λ*_max_, while maintaining values consistently higher than the blank at all concentrations. This behavior is consistent with partial retention of ordered helical structures. Notably, CRP-UAAE consistently exhibited higher *λ*_max_ values than CRP-CAE across the tested NaOH concentration range, particularly at 0.3–0.4 mol/L, indicating greater conformational stability under alkaline conditions. The *λ*_max_ of UAAE-CRP remained 3–5 nm higher than that of CAE-CRP throughout the alkali gradient, suggesting that ultrasonic treatment more effectively preserves ordered secondary structures than prolonged thermal–alkaline exposure. Because helical conformations, especially triple-helix–like structures, are frequently associated with enhanced immunomodulatory and antioxidant activities in fungal polysaccharides [6], [43], the superior conformational retention observed in UAAE-derived CRP is consistent with its improved bioactivity profile.

#### 3.5.10 Thermal stability

Thermal stability was evaluated to assess heat resistance and compositional integrity of polysaccharides extracted by UAAE and CAE. The TGA/DTG profiles and thermodynamic parameters (Fig. 9C–D) revealed distinct thermal behaviors. Both CRP-UAAE and CRP-CAE exhibited four decomposition stages, while total mass loss at 600 °C was lower for CRP-UAAE (55.91%) than for CRP-CAE (61.25%), indicating enhanced thermal stability or reduced volatile content. The principal decomposition temperature (*T*_3_), corresponding to polysaccharide backbone degradation, was 237.41 °C for CRP-UAAE, which was 22 °C higher than that of CRP-CAE (215.27 °C). The final decomposition stage also occurred at a higher temperature for CRP-UAAE (355.50 °C versus 319.02 °C). Thermodynamic analysis (Table S3) showed a lower dehydration enthalpy (Δ*H*_12_ = 451.60 J/g) for CRP-UAAE than for CRP-CAE (546.32 J/g), reflecting differences in water-binding capacity. CRP-UAAE exhibited stronger exothermic transitions during Δ*H*_23_ (−70.64 J/g) and Δ*H*_34_ (−116.27 J/g), suggesting higher energy density or more stable glycosidic linkages. DTG curves supported these observations, with CRP-UAAE displaying sharper and more defined peaks in the 200–300 °C range compared with broader transitions for CRP-CAE. The superior thermal stability of CRP-UAAE is consistent with preservation of higher-molecular-weight fractions and selective retention of stable glycosidic bonds, supporting its suitability for applications involving elevated processing temperatures.

#### 3.5.11 Particle size and zeta potential

Dynamic light scattering and zeta potential measurements were employed to evaluate colloidal properties of CRP-UAAE and CRP-CAE. As shown in Fig. 9E, CRP-CAE exhibited a monomodal particle size distribution with a Z-average diameter of 373.3 nm and a dominant peak at 225.4 nm, together with a polydispersity index of 0.564, indicating moderate size heterogeneity. In contrast, CRP-UAAE displayed a bimodal distribution with a smaller Z-average diameter of 224.7 nm, featuring peaks at 196.8 nm and 44.2 nm, and a lower PDI of 0.423, reflecting improved uniformity and reduced aggregation. The high proportion of small particles in CRP-UAAE confirms effective cavitation-induced fragmentation of large aggregates and formation of more homogeneous dispersions. Zeta potential analysis (Table 4) further distinguished the samples, with CRP-CAE exhibiting a single negatively charged population and CRP-UAAE showing a heterogeneous charge distribution with higher conductivity. This heterogeneity likely reflects the presence of polysaccharide fractions bearing diverse functional groups and uronic acid residues, consistent with the more complex structural profile generated under ultrasound-assisted extraction [31]. Both samples exhibited zeta potentials characteristic of moderate colloidal stability, while the smaller particle size, lower PDI, and broader charge distribution of CRP-UAAE indicate improved dispersibility, solubility, and potential bioavailability.

#### 3.5.12 Rheological properties

Rheological properties of CRPs extracted by UAAE and CAE were evaluated by steady-shear, amplitude sweep, and frequency sweep measurements (Fig. 9F–H). Both samples exhibited non-Newtonian shear-thinning behavior over the shear rate range of 0.1–1000 s⁻^1^, with viscosity decreasing as shear rate increased. At low shear rates (0.1–1 s⁻^1^), CRP-CAE showed higher viscosity than CRP-UAAE, whereas at higher shear rates (>100 s⁻^1^), the viscosities converged (∼0.001–0.003 Pa·s), indicating reduced flow resistance under strong shear. Oscillatory measurements revealed differences in viscoelastic behavior. In amplitude sweeps, CRP-CAE exhibited a crossover from viscous dominance (G″ > G′) to elastic dominance (G′ > G″), whereas CRP-UAAE maintained G′ > G″ across the tested strain range, indicating a predominantly elastic, gel-like network. Frequency sweep results further differentiated the samples. At low frequencies (<0.04 Hz), CRP-UAAE exhibited higher G′ values than CRP-CAE, indicating greater long-range elasticity. At higher frequencies, CRP-UAAE showed lower G′ values, suggesting reduced resistance to rapid deformation, likely due to cavitation-induced reduction in entanglement density. For both samples, G″ remained lower than G′ across the measured frequency range, confirming dominant elastic behavior. These rheological characteristics indicate that ultrasonic treatment alters molecular architecture and viscoelastic dynamics. Lower viscosity of CRP-UAAE is consistent with partial depolymerization and reduced chain entanglement under cavitation [44], [45]. At the same time, stable G′ > G″ behavior suggests formation of a cohesive network, potentially driven by rearranged hydrogen bonding and exposure of interactive groups [46]. The frequency-dependent G′ response indicates that CRP-UAAE exhibit strong static elasticity but weaker high-frequency resistance, consistent with flexible yet structured polymer networks [37]. These features indicate that ultrasonic extraction modifies both microstructure and rheology, yielding polysaccharides with improved flowability and stable low-frequency elasticity, which is advantageous for food, biomedical, and controlled-release applications.

### 3.6 Antioxidant activity analysis

#### 3.6.1 ABTS radical scavenging activity

**Fig. 10:**
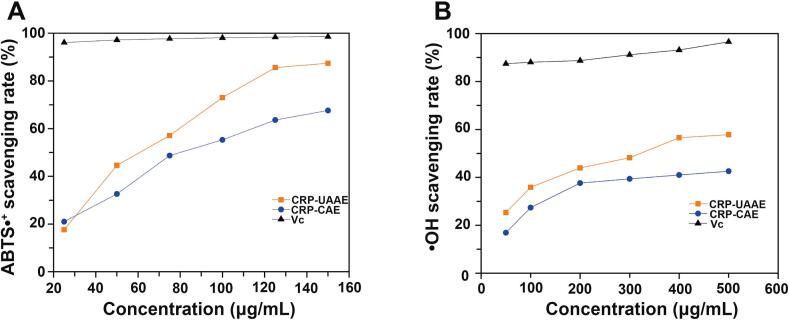
Free radical scavenging activities of CRPs. (A) ABTS radical scavenging activity of polysaccharide samples at different concentrations. (B) Hydroxyl radical scavenging activity of polysaccharide samples at different concentrations. CRP-UAAE, polysaccharides extracted by UAAE; CRP-CAE, polysaccharides extracted by CAE; Vc, vitamin C (positive control). Data are presented as mean ± SD (n = 3).

ABTS•⁺ scavenging capacities of CRP extracted by UAAE and CAE were evaluated using ascorbic acid (Vc) as a reference antioxidant (Fig. 10A). Vc was included solely as a benchmark, and direct equivalence of IC_50_ values was not anticipated because small-molecule antioxidants and polysaccharides operate through distinct mechanisms. Both samples exhibited pronounced concentration-dependent scavenging activity over the range of 25–150 μg/mL, displaying typical dose–response behavior. At 150 μg/mL, CRP-UAAE reached a scavenging rate of 87.4%, substantially higher than that of CRP-CAE (67.6%), indicating superior antioxidant capacity for the ultrasound-assisted extract. The corresponding IC_50_ were 55.7 μg/mL for CRP-UAAE and 81.4 μg/mL for CRP-CAE, representing a 31.6% reduction and confirming enhanced radical-scavenging efficiency under UAAE. Although both polysaccharide extracts were less potent than Vc (IC_50_ = 2.25 μg/mL), CRP-UAAE and CRP-CAE showed approximately 24-fold and 35-fold lower apparent potency, respectively, which is consistent with the inherently lower reactivity and diffusion-limited kinetics of macromolecular antioxidants. The enhanced ABTS•⁺ scavenging activity of CRP-UAAE is attributed to structural differences comprehensively analyzed in Section 3.6.3.

#### 3.6.2 Hydroxyl radical scavenging activity

The hydroxyl radical (•OH) scavenging activity of CRPs extracted by UAAE and CAE was evaluated using a Fenton reaction system, with Vc as the reference antioxidant (Fig. 10B). Both samples exhibited concentration-dependent scavenging behavior over the range of 0.05–0.5 mg/mL, with CRP-UAAE consistently showing higher activity than CRP-CAE. At 0.5 mg/mL, CRP-UAAE achieved a scavenging rate of 70.2%, whereas CRP-CAE reached 62.6%. The IC_50_ value of CRP-UAAE (0.17 mg/mL) was markedly lower than that of CRP-CAE (0.24 mg/mL), indicating improved effectiveness against highly reactive oxygen species. Hydroxyl radicals attack biomolecules primarily through hydrogen abstraction and addition reactions, and polysaccharides scavenge •OH mainly by donating hydrogen atoms from hydroxyl groups to generate more stable radical intermediates [47], [48]. The structural basis for the enhanced •OH-scavenging capacity of CRP-UAAE is analyzed comprehensively in Section 3.6.3. Compared with ABTS•⁺ scavenging, both CRPs were less effective against •OH, with IC_50_ values approximately threefold higher, reflecting the extreme reactivity and short lifetime of •OH. These findings indicate radical-type dependence of antioxidant efficacy, with higher activity toward moderately reactive species such as ABTS•⁺.

#### 3.6.3 Structure–activity relationship for enhanced antioxidant activity

Molecular weight distribution serves as the primary structural determinant of radical-scavenging efficiency. The bimodal distribution of CRP-UAAE, comprising preserved high-molecular-weight chains (7.54 × 10^5^ Da, 45.68%) combined with a dominant moderate-molecular-weight fraction (1.32 × 10^4^ Da, 54.32%), provides a favorable architectural basis for enhanced antioxidant activity. This distribution integrates the high solubility and diffusion capacity associated with moderate-molecular-weight chains with the multivalent interaction potential of extended polymer backbones. High-molecular-weight fractions contribute abundant hydroxyl groups distributed along elongated chains, enabling multiple, cooperative radical-scavenging interactions and effective stabilization of radical species [15]. Moderate-molecular-weight components are better aligned with the kinetic requirements of short-lived radicals such as •OH, supporting efficient radical interception within limited lifetimes [49], [50]. In contrast, CRP-CAE exhibited excessive depolymerization, characterized by a dominant low-molecular-weight fraction at 1.43 × 10^3^ Da (51.11%), which constrains cooperative interactions and multivalent scavenging behavior. This structural limitation is consistent with its inferior antioxidant performance, reflected by higher IC_5_*_0_* values for both ABTS•⁺ (81.4 vs. 55.7 μg/mL) and •OH (0.24 vs. 0.17 mg/mL).

Conformational ordering represents a secondary but functionally significant contributor to antioxidant activity. CRP-UAAE exhibited superior retention of ordered helical conformations, as evidenced by a larger Congo red bathochromic shift (Δ*λ*_max_ = 5–6 nm, compared with minimal shifts for CRP-CAE) and enhanced thermal stability, indicated by a higher decomposition temperature (237.41 °C vs. 215.27 °C). Ordered helical architectures stabilize polysaccharide chains while maintaining surface hydroxyl groups in accessible spatial configurations, thereby supporting efficient hydrogen donation and limiting conformational collapse that could obscure reactive sites [6], [51]. The coexistence of preserved molecular weight and maintained conformational order establishes a structurally favorable framework for antioxidant function.

The magnitude of antioxidant enhancement further exhibited clear radical-type specificity, with IC_50_ reductions of 31.6% for ABTS•⁺ and 29.2% for •OH. Such differentiation aligns with the distinct physicochemical characteristics of the radicals, whereby ABTS•⁺ scavenging is primarily associated with molecular weight distribution, while •OH scavenging is more closely linked to conformational accessibility and the density of hydroxyl-rich functional domains [52], [53]. These findings demonstrate that the enhanced antioxidant activity of CRP-UAAE originates from the synergistic effects of a balanced molecular weight distribution, retained ordered conformations, and favorable structural features, supporting the potential application of UAAE-derived polysaccharides in functional food and nutraceutical systems where antioxidant capacity is a critical quality attribute.

## 4 Conclusion

This study integrates support vector regression–based process optimization with ultrasonic intensification to achieve targeted extraction of bioactive fungal polysaccharides. Polysaccharides from *C. rutilus* were extracted using an SVR-optimized UAAE approach. Guided by single-factor experiments, the SVR model was applied to optimize six critical process parameters and showed higher predictive accuracy than quadratic RSM, increasing the testing *R*2 from 0.8751 to 0.9027 and reducing RMSE by 11.7%. Under the optimal UAAE conditions, comprising ultrasonic temperature 56.5 °C, soaking time 138 min, ultrasonic time 35 min, liquid–solid ratio 26 mL/g, NaOH concentration 0.55 mol/L, and ultrasonic power 325 W, the extraction yield reached 20.09 ± 0.10%, representing a 53.7% improvement compared with CAE. HPGPC revealed two dominant molecular weight fractions at 7.54 × 10^5^ and 1.32 × 10^4^ Da. Structural characterization demonstrates that UAAE more effectively preserves ordered helical conformations, supported by concordant evidence from Congo red assay, XRD, TGA, and AFM analyses. Polysaccharides obtained by UAAE exhibited significantly enhanced antioxidant activity in ABTS and hydroxyl radical scavenging assays compared with CAE-derived products.

Beyond *C. rutilus*, this work highlights the broader methodological value of combining data-driven SVR optimization with ultrasonic process intensification as a general strategy for targeted extraction of bioactive polysaccharides. This integration enables rational process design, precise control of structural integrity, and concurrent enhancement of efficiency and sustainability, providing a transferable framework for advanced bioprocessing of functional natural products.

## CRediT authorship contribution statement

**Xiaorong Zhang:** Writing – original draft, Software, Conceptualization. **Yufan Zeng:** Investigation, Formal analysis. **Jiehan Zhang:** Validation, Resources. **Weiye Duan:** Visualization, Investigation. **Xinhua Ao:** Investigation, Data curation. **Huizhu Wang:** Supervision, Project administration, Methodology. **Shuai Chen:** Writing – review & editing, Supervision, Resources, Funding acquisition, Conceptualization.

## Declaration of competing interest

The authors declare that they have no known competing financial interests or personal relationships that could have appeared to influence the work reported in this paper.

## Appendix A Supplementary data

Supplementary Data 1 Comparative model performance analyses (error distribution, residuals, and prediction accuracy) of SVR and RSM models (Fig. S1), partial dependence plots illustrating the effects of key extraction variables on CRP yield (Fig. S2), and temporal validation of model predictions (Fig. S3). Additionally, detailed CCD experimental design and corresponding extraction yields (Table S1), relative importance ranking of extraction parameters (Table S2), and thermal transition properties of raw materials and extracted polysaccharides (Table S3) are provided.
