# Supplementary material for: Support vector Regression-Optimized Ultrasonic-Assisted alkaline extraction of Chroogomphus rutilus Polysaccharides: Enhanced yield and preservation of helical conformations
Source: Ultrason Sonochem. 2026 May 3;129:107879. doi: 10.1016/j.ultsonch.2026.107879 (PMC13156778; doi:10.1016/j.ultsonch.2026.107879)
Supplement: Supplementary Data 1 — Comparative model performance analyses (error distribution, residuals, and prediction accuracy) of SVR and RSM models (Fig. S1), partial dependence plots illustrating the effects of key extraction variables on CRP yield (Fig. S2), and temporal validation of model predictions (Fig. S3). Additionally, detailed CCD experimental design and corresponding extraction yields (Table S1), relative importance ranking of extraction parameters (Table S2), and thermal transition properties of raw materials and extracted polysaccharides (Table S3) are provided. [file mmc1.docx]

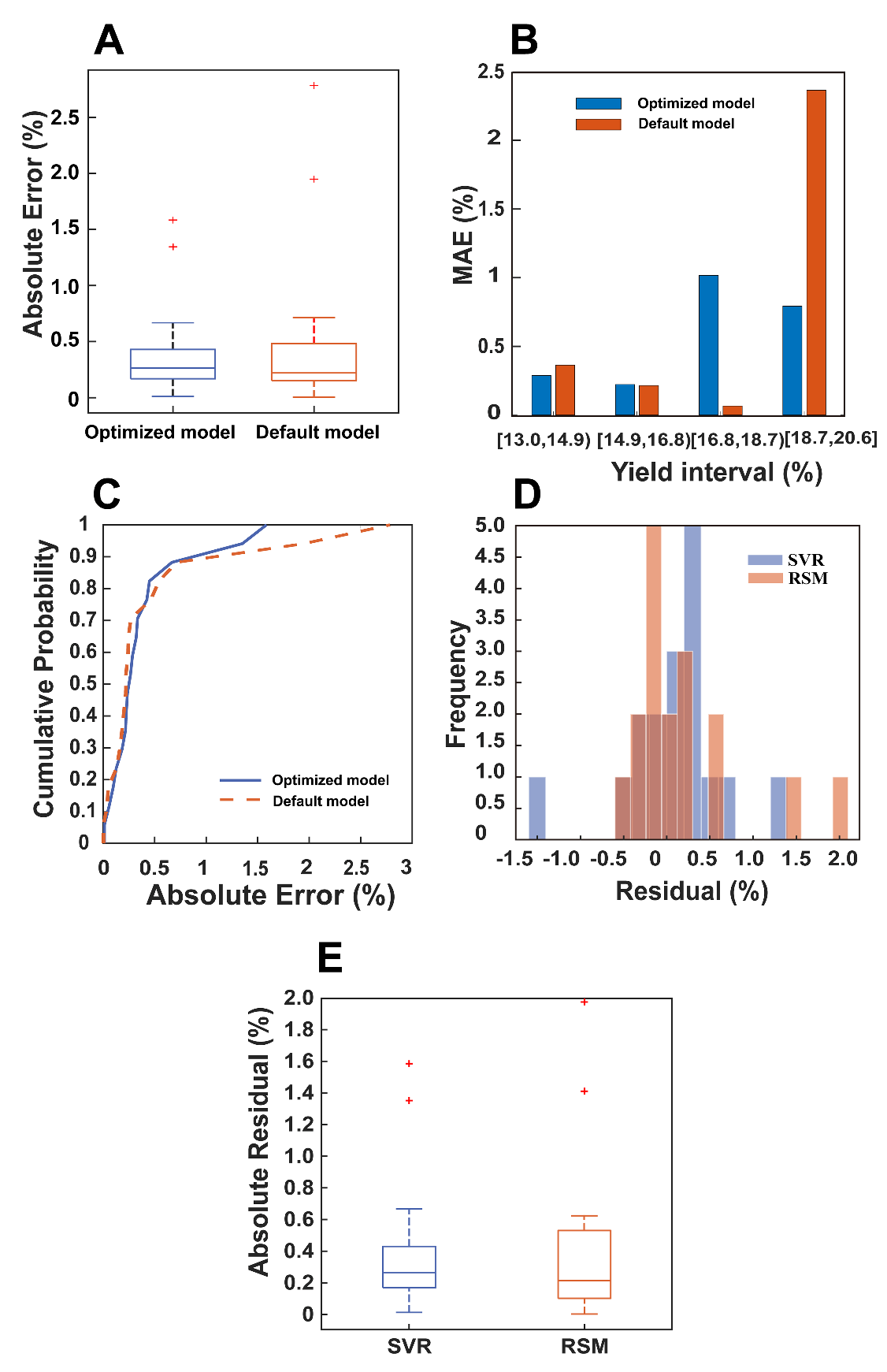


**Fig. S1. Comparative error and residual analyses of SVR and RSM models for CRP yield prediction. (A) Box-plot comparison of absolute errors between the optimized and baseline SVR models; (B) variation of mean absolute error (MAE) across different yield intervals; (C) cumulative distribution functions (CDFs) of absolute prediction errors; (D) residual distribution comparison between SVR and RSM models; and (E) box-plot comparison of absolute residuals.**

**
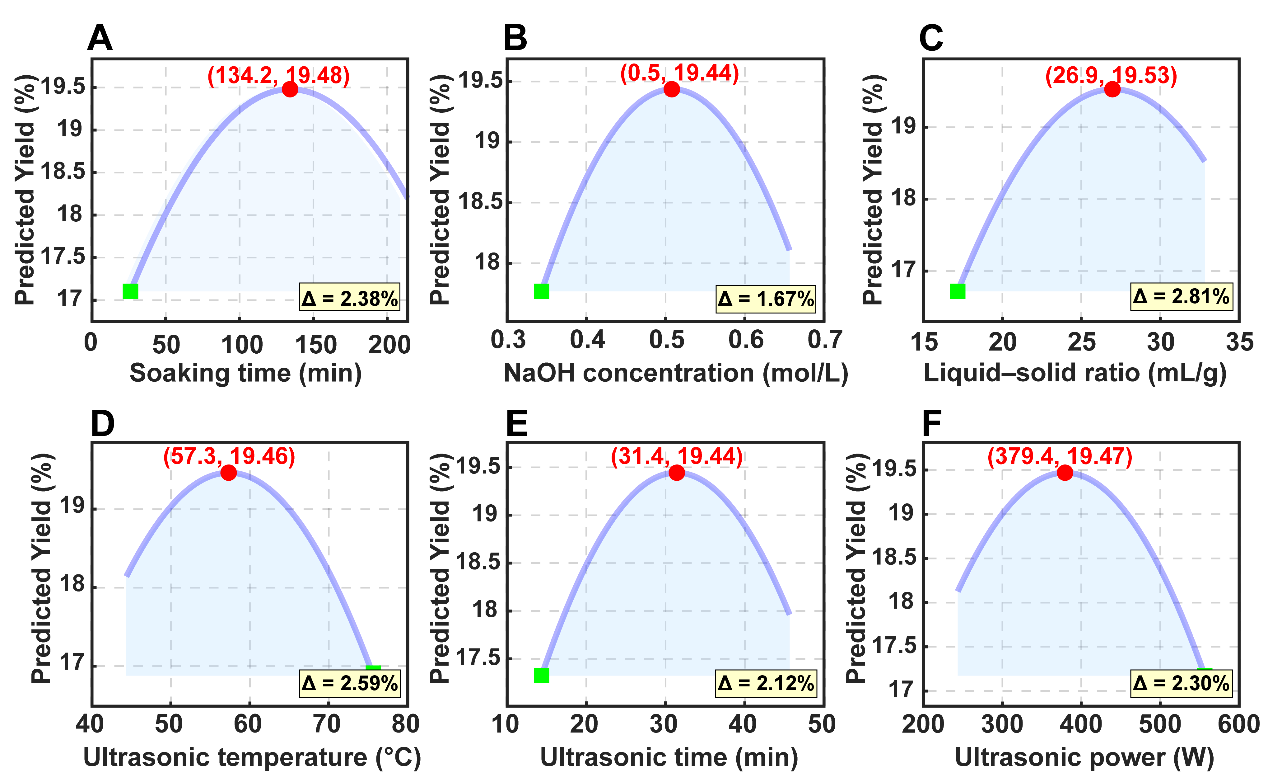
**

**Fig. S2. Partial dependence plots depicting the marginal effects of individual variables on the predicted CRP yield. (A) Soaking time; (B) NaOH concentration; (C) Liquid–solid ratio; (D) Ultrasonic temperature; (E) Ultrasonic time; (F) Ultrasonic power. Shaded regions denote 95 % confidence intervals.**

**
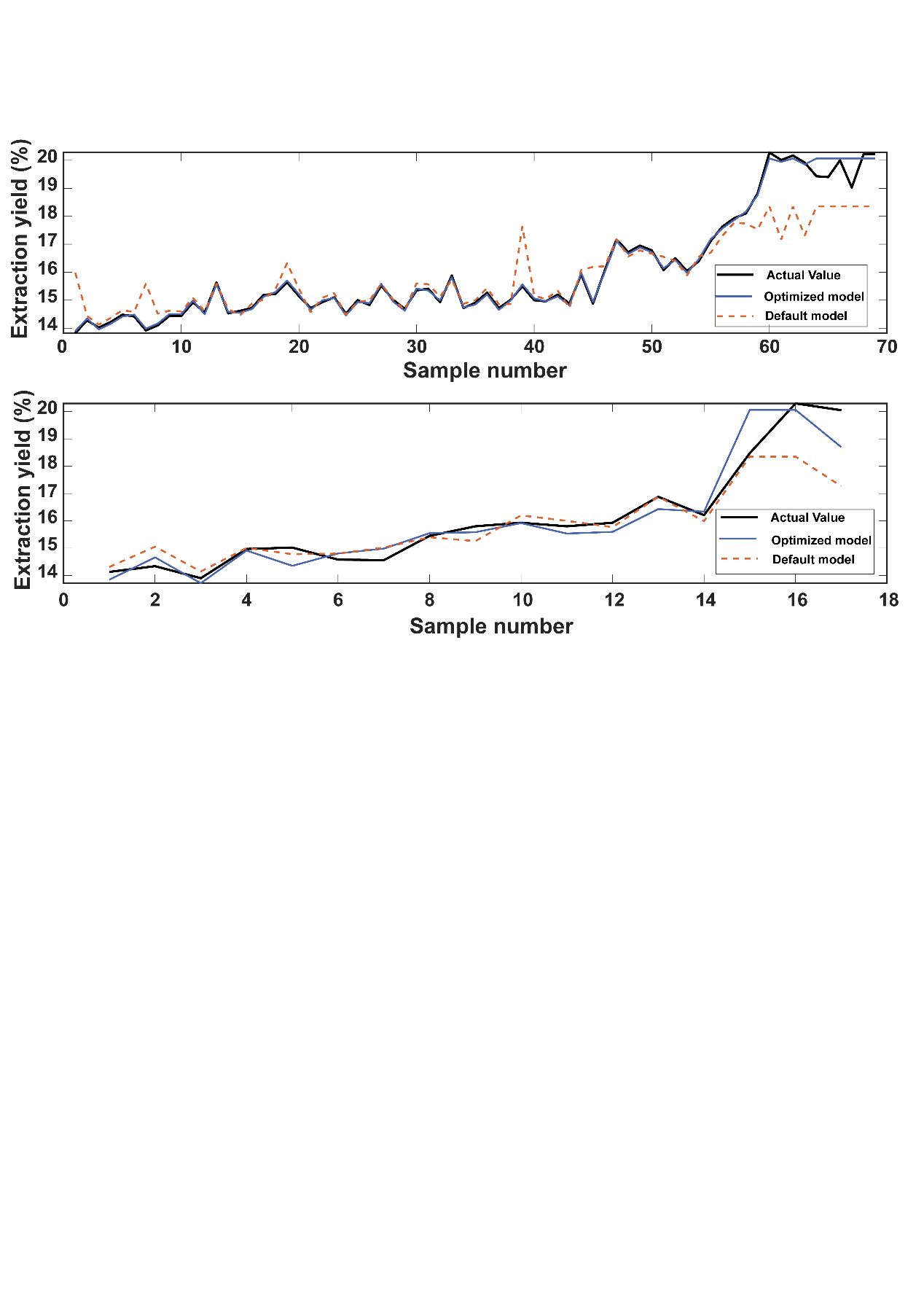
**

**Fig. S3. Temporal comparison of experimental and predicted extraction yields for the optimized and baseline SVR models. (A) Training sequence (n = 69) demonstrating strong predictive agreement (R² = 0.9907). (B) Testing sequence (n = 17) highlighting the superior predictive fidelity of the optimized SVR model in the high-yield region.**

**TableS1. CCD experimental design and corresponding CRP extraction yields.**

| No. | *X_1_* | *X_2_* | *X_3_* | *X_4_* | *X_5_* | *X_6_* | *Y* (%) |
| --- | --- | --- | --- | --- | --- | --- | --- |
|  | Soaking time  (min) | NaOH concentration  (mol/L) | Liquid-solid ratio  (mL/g) | Ultrasonic temperature  (°C) | Ultrasonic time  (min) | Ultrasonic power  (W) |  |
| 1 | 60 | 0.6 | 30 | 70 | 40 | 500 | 14.72 |
| 2 | 60 | 0.6 | 20 | 50 | 20 | 500 | 14.42 |
| 3 | 120 | 0.5 | 32.8254 | 60 | 30 | 400 | 15.50 |
| 4 | 180 | 0.6 | 20 | 70 | 40 | 300 | 15.80 |
| 5 | 60 | 0.4 | 30 | 70 | 40 | 500 | 14.72 |
| 6 | 180 | 0.4 | 20 | 70 | 20 | 300 | 14.62 |
| 7 | 120 | 0.5 | 25 | 60 | 30 | 400 | 20.28 |
| 8 | 60 | 0.4 | 20 | 70 | 20 | 500 | 14.22 |
| 9 | 120 | 0.5 | 25 | 60 | 30 | 400 | 20.30 |
| 10 | 180 | 0.6 | 20 | 50 | 20 | 500 | 14.56 |
| 11 | 60 | 0.4 | 20 | 70 | 20 | 300 | 13.90 |
| 12 | 60 | 0.6 | 30 | 70 | 20 | 300 | 15.02 |
| 13 | 120 | 0.5 | 25 | 60 | 30 | 556.508 | 17.11 |
| 14 | 180 | 0.4 | 20 | 70 | 40 | 500 | 14.97 |
| 15 | 180 | 0.6 | 30 | 50 | 40 | 500 | 15.64 |
| 16 | 60 | 0.4 | 30 | 50 | 40 | 500 | 16.04 |
| 17 | 180 | 0.6 | 20 | 70 | 20 | 300 | 14.44 |
| 18 | 180 | 0.6 | 20 | 70 | 20 | 500 | 14.96 |
| 19 | 180 | 0.4 | 30 | 70 | 20 | 500 | 14.91 |
| 20 | 60 | 0.6 | 30 | 50 | 20 | 500 | 15.15 |
| 21 | 60 | 0.6 | 20 | 70 | 20 | 300 | 14.02 |
| 22 | 60 | 0.4 | 30 | 50 | 40 | 300 | 16.78 |
| 23 | 120 | 0.5 | 25 | 75.6508 | 30 | 400 | 13.83 |
| 24 | 60 | 0.4 | 30 | 70 | 40 | 300 | 14.34 |
| 25 | 120 | 0.343492 | 25 | 60 | 30 | 400 | 16.08 |
| 26 | 180 | 0.4 | 20 | 70 | 40 | 300 | 15.00 |
| 27 | 120 | 0.5 | 25 | 60 | 30 | 400 | 19.43 |
| 28 | 180 | 0.4 | 30 | 50 | 40 | 500 | 16.06 |
| 29 | 60 | 0.4 | 20 | 70 | 40 | 300 | 14.13 |
| 30 | 60 | 0.6 | 30 | 50 | 40 | 300 | 16.95 |
| 31 | 120 | 0.5 | 25 | 60 | 14.3492 | 400 | 16.40 |
| 32 | 60 | 0.4 | 30 | 50 | 20 | 300 | 16.72 |
| 33 | 180 | 0.6 | 30 | 50 | 20 | 300 | 17.63 |
| 34 | 180 | 0.4 | 30 | 70 | 40 | 300 | 15.41 |
| 35 | 180 | 0.6 | 30 | 70 | 20 | 500 | 15.27 |
| 36 | 60 | 0.4 | 20 | 50 | 20 | 500 | 14.52 |
| 37 | 26.0949 | 0.5 | 25 | 60 | 30 | 400 | 14.88 |
| 38 | 60 | 0.4 | 30 | 50 | 20 | 500 | 15.88 |
| 39 | 120 | 0.5 | 25 | 60 | 30 | 400 | 19.03 |
| 40 | 120 | 0.5 | 25 | 60 | 30 | 243.492 | 20.05 |
| 41 | 60 | 0.6 | 30 | 70 | 20 | 500 | 15.02 |
| 42 | 180 | 0.6 | 20 | 50 | 20 | 300 | 15.00 |
| 43 | 180 | 0.6 | 30 | 70 | 40 | 300 | 15.92 |
| 44 | 180 | 0.4 | 20 | 50 | 20 | 300 | 14.83 |
| 45 | 60 | 0.6 | 30 | 50 | 20 | 300 | 16.50 |
| 46 | 60 | 0.6 | 20 | 70 | 20 | 500 | 14.88 |
| 47 | 120 | 0.5 | 25 | 60 | 30 | 400 | 20.23 |
| 48 | 180 | 0.6 | 30 | 50 | 40 | 300 | 17.93 |
| 49 | 60 | 0.6 | 30 | 70 | 40 | 300 | 14.93 |
| 50 | 180 | 0.4 | 30 | 70 | 40 | 500 | 15.10 |
| 51 | 120 | 0.5 | 25 | 60 | 30 | 400 | 19.40 |
| 52 | 60 | 0.4 | 30 | 70 | 20 | 500 | 14.48 |
| 53 | 60 | 0.4 | 20 | 50 | 40 | 300 | 14.72 |
| 54 | 60 | 0.6 | 20 | 70 | 40 | 500 | 15.01 |
| 55 | 180 | 0.4 | 30 | 50 | 20 | 500 | 15.80 |
| 56 | 60 | 0.4 | 20 | 50 | 40 | 500 | 14.44 |
| 57 | 60 | 0.6 | 20 | 70 | 40 | 300 | 14.28 |
| 58 | 120 | 0.5 | 17.1746 | 60 | 30 | 400 | 13.92 |
| 59 | 180 | 0.6 | 30 | 50 | 20 | 500 | 15.93 |
| 60 | 180 | 0.4 | 20 | 50 | 40 | 300 | 15.52 |
| 61 | 60 | 0.6 | 30 | 50 | 40 | 500 | 15.35 |
| 62 | 180 | 0.4 | 30 | 70 | 20 | 300 | 15.20 |
| 63 | 180 | 0.4 | 20 | 70 | 20 | 500 | 14.56 |
| 64 | 180 | 0.6 | 20 | 50 | 40 | 500 | 15.22 |
| 65 | 60 | 0.6 | 20 | 50 | 40 | 500 | 14.58 |
| 66 | 180 | 0.6 | 20 | 70 | 40 | 500 | 15.45 |
| 67 | 120 | 0.5 | 25 | 60 | 30 | 400 | 20.17 |
| 68 | 120 | 0.5 | 25 | 60 | 30 | 400 | 20.00 |
| 69 | 180 | 0.4 | 30 | 50 | 40 | 300 | 17.17 |
| 70 | 213.905 | 0.5 | 25 | 60 | 30 | 400 | 18.82 |
| 71 | 60 | 0.4 | 30 | 70 | 20 | 300 | 15.19 |
| 72 | 60 | 0.4 | 20 | 70 | 40 | 500 | 14.72 |
| 73 | 60 | 0.6 | 20 | 50 | 40 | 300 | 14.92 |
| 74 | 120 | 0.5 | 25 | 60 | 30 | 400 | 18.48 |
| 75 | 120 | 0.5 | 25 | 60 | 30 | 400 | 20.21 |
| 76 | 180 | 0.4 | 20 | 50 | 40 | 500 | 14.93 |
| 77 | 120 | 0.656508 | 25 | 60 | 30 | 400 | 20.01 |
| 78 | 120 | 0.5 | 25 | 60 | 45.6508 | 400 | 19.92 |
| 79 | 180 | 0.6 | 30 | 70 | 20 | 300 | 15.93 |
| 80 | 120 | 0.5 | 25 | 44.3492 | 30 | 400 | 18.10 |
| 81 | 180 | 0.4 | 20 | 50 | 20 | 500 | 14.53 |
| 82 | 60 | 0.6 | 20 | 50 | 20 | 300 | 14.10 |
| 83 | 180 | 0.4 | 30 | 50 | 20 | 300 | 16.88 |
| 84 | 180 | 0.6 | 30 | 70 | 40 | 500 | 15.63 |
| 85 | 60 | 0.4 | 20 | 50 | 20 | 300 | 14.69 |
| 86 | 180 | 0.6 | 20 | 50 | 40 | 300 | 16.21 |

**Table S2. Relative importance ranking of extraction parameters.**

| **Rank** | **Variable** | **Permutation Importance** | **SV Contribution** | **PD Range** | **Combined Score** |
| --- | --- | --- | --- | --- | --- |
| 1 | Ultrasonic time (min) | 1.0000 | 0.2847 | 0.7544 | 0.7598 |
| 2 | Liquid–solid ratio (mL/g) | 0.7800 | 0.2234 | 1.0000 | 0.6959 |
| 3 | Soaking time (min) | 0.6500 | 0.1756 | 0.8470 | 0.5807 |
| 4 | NaOH concentration (mol/L) | 0.5200 | 0.1423 | 0.5943 | 0.4442 |
| 5 | Ultrasonic power (W) | 0.3600 | 0.1012 | 0.8185 | 0.4099 |
| 6 | Temperature (°C) | 0.1800 | 0.0728 | 0.9217 | 0.3386 |

**Table S3.** **Thermal transition temperatures and enthalpy changes of CR and CRPs.**

|  | ***T***_1_(℃) | ***T***_2_(℃) | ***T***_3_(℃) | ***T***_4_(℃) | Δ***H***_12_(J/g) | Δ***H***_23_(J/g) | Δ***H***_34_(J/g) |
| --- | --- | --- | --- | --- | --- | --- | --- |
| CR | 69.43 | 114.38 | 233.39 | 499.94 | 159.09 | -183.74 | 3559.0 |
| CR-UAAE | 68.57 | 166.01 | 244.38 | 480.87 | 690.51 | -184.81 | 3586.1 |
| CR-CAE | 71.38 | 165.94 | 236.38 | 471.78 | 589.17 | -188.20 | 2609.1 |
| CRP-UAAE | 61.95 | 143.15 | 237.41 | 355.50 | 451.60 | -70.64 | -116.27 |
| CRP-CAE | 63.11 | 144.74 | 215.27 | 319.02 | 546.32 | -55.58 | -65.81 |
